# Disorder-specific neurodynamic features in schizophrenia inferred by neurodynamic embedded contrastive variational autoencoder model

**DOI:** 10.1038/s41398-024-03200-7

**Published:** 2024-12-18

**Authors:** Chaoyue Ding, Yuqing Sun, Kunchi Li, Sangma Xie, Hao Yan, Peng Li, Jun Yan, Jun Chen, Huiling Wang, Huaning Wang, Yunchun Chen, Yongfeng Yang, Luxian Lv, Hongxing Zhang, Lin Lu, Dai Zhang, Yaojing Chen, Zhanjun Zhang, Tianzi Jiang, Bing Liu

**Affiliations:** 1 School of Artificial Intelligence, University of Chinese Academy of Sciences, Beijing, 100049 China; 2 Brainnetome Center, Institute of Automation, Chinese Academy of Sciences, Beijing, 100190 China; 3 State Key Laboratory of Cognitive Neuroscience and Learning, Beijing Normal University, Beijing, 100875 China; 4 Institute of Biomedical Engineering and Instrumentation, School of Automation, Hangzhou Dianzi University, Hangzhou, 310018 China; 5 Institute of Mental Health, Peking University Sixth Hospital, Beijing, 100191 China; 6 Key Laboratory of Mental Health, Ministry of Health, and National Clinical Research Center for Mental Disorders (Peking University Sixth Hospital), Beijing, 100191 China; 7 Department of Radiology, Renmin Hospital of Wuhan University, Wuhan, 430060 China; 8 Department of Psychiatry, Renmin Hospital of Wuhan University, Wuhan, 430060 China; 9 Department of Psychiatry, Xijing Hospital, The Fourth Military Medical University, Xi’an, 710032 China; 10 Department of Psychiatry, Henan Mental Hospital, The Second Affiliated Hospital of Xinxiang Medical University, Xinxiang, 453002 China; 11 Henan Key Lab of Biological Psychiatry of Xinxiang Medical University, International Joint Research Laboratory for Psychiatry and Neuroscience of Henan, Xinxiang, 453002 China; 12 IDG/McGovern Institute for Brain Research, Beijing Normal University, Beijing, 100875 China; 13 Research Center for Augmented Intelligence, Zhejiang Lab, Hangzhou, 311100 China; 14 Innovation Academy for Artificial Intelligence, Chinese Academy of Sciences, Beijing, 100190 China; 15 Chinese Institute for Brain Research, Beijing, 102206 China

**Keywords:** Schizophrenia, Molecular neuroscience

## Abstract

Neurodynamic models that simulate how micro-level alterations propagate upward to impact macroscopic neural circuits and overall brain function may offer valuable insights into the pathological mechanisms of schizophrenia (SCZ). In this study, we integrated a neurodynamic model with the classical Contrastive Variational Autoencoder (CVAE) to extract and evaluate macro-scale SCZ-specific features, including subject-level, region-level parameters, and time-varying states. Firstly, we demonstrated the robust fitting of the model within our multi-site dataset. Subsequently, by employing representational similarity analysis and a deep learning classifier, we confirmed the specificity and disorder-related information capturing ability of SCZ-specific features. Moreover, analysis of the attractor characteristics of the neurodynamic system revealed significant differences in attractor space patterns between SCZ-specific states and shared states. Finally, we utilized Partial Least Squares (PLS) regression to examine the multivariate mapping relationship between SCZ-specific features and symptoms, identifying two sets of correlated modes implicating unique molecular mechanisms: one mode corresponding to negative and general symptoms, and another mode corresponding to positive symptoms. Our results provide valuable insights into disorder-specific neurodynamic features and states associated with SCZ, laying the foundation for understanding the intricate pathophysiology of this disorder.

## Introduction

Schizophrenia (SCZ) is a severe psychiatric disorder characterized by distinct alterations across multiple biological levels and scales. At the molecular level, transcriptomic profiling has revealed changes in the expression of various genes involved in regulating synaptic function and signal transduction within the cortical regions of SCZ patients [1]. At the microcircuit level, histological studies have shown that altered synaptic density in SCZ brains may disrupt the balance between synaptic excitation and inhibition [2]. On a macro-scale, noninvasive neuroimaging techniques have identified numerous spatiotemporal dynamic abnormalities associated with SCZ [3, 4]. To elucidate the micro-level mechanisms driving these spatiotemporal disruptions, computational models that incorporate microstructural parameters have been employed. These models provide a framework for exploring how micro-level alterations propagate through the brain, ultimately impacting macroscopic neural circuits and overall brain function [5, 6].

Among them, biophysically based neurodynamic network models have gained increasing attention due to their effective fitting performance on neuroimaging or EEG data with high spatial or temporal resolution [7]. In these models, the local dynamics of each brain region are represented by neural mass models, with interactions between brain regions constrained by structural connectivity (SC), and model parameters optimized using robust metrics such as functional connectivity (FC) [8]. Previous studies applying these neurodynamic models to SCZ datasets have confirmed associations between neurodynamic parameters (such as E-I ratio, global coupling G) of SCZ and disorder factors related to information interaction [9], as well as alterations in global brain signals [10]. However, given the complexity of brain spatiotemporal dynamics and the extensive variability among individuals, two significant challenges remain in revealing the intricate functional organization and spatiotemporal dynamics in SCZ brains and establishing associations with symptoms.

Firstly, due to computational expense, most SCZ studies have focused on simulating neurodynamic features at the group level to explore the dynamic mechanisms underlying the specific dysfunctions associated with the disorder [5, 9, 10]. Given the extensive heterogeneity in symptoms, cognition, and antipsychotic drug response in SCZ [5, 11], investigating individualized neurodynamic mechanisms is crucial for a deeper understanding of SCZ pathophysiology and for developing precise diagnostic and therapeutic strategies. A recent breakthrough study [12], introduced the deep variational autoencoder (VAE) method to infer individualized model parameters of the neural mass model from fMRI data, reliably identifying specific parameters for each subject and each brain region with a clear and distinct role in the dynamics. This approach holds the potential to unravel the complex, individualized spatiotemporal dynamics in the brains of SCZ patients.

The second major challenge lies in extracting disorder-specific abnormal patterns from neuroimaging data. Establishing detailed mapping relationships between clinical symptoms and abnormal neuroimaging phenotypes, as well as conducting in-depth research on the neurobiological basis of SCZ phenotype diversity, has become a focus of recent research [13]. For instance, recent neuroimaging-clinic correlation analyses have revealed detailed relationships between clinical symptom heterogeneity and neuroanatomical abnormalities in anatomically defined subgroups [14]. However, neuroimaging features or biomarkers directly extracted from MRI data in SCZ often lack disorder specificity, resulting in many normal individuals being misclassified into a certain SCZ subtype or exhibiting neuroimaging features similar to SCZ. Paradoxically, symptom scores or descriptions commonly used for SCZ patients are often not applicable to healthy individuals. For instance, most Positive and Negative Syndrome Scale (PANSS) symptoms do not appear in healthy individuals, who typically score zero [15]. Additionally, many individual differences in neuroimaging are driven by genetic and environmental factors unrelated to SCZ, making it easy to introduce confounding factors such as site, gender, and age when directly using extracted SCZ features [16]. A previous study utilized Contrastive Variational Autoencoder (CVAE) to distinguish autism spectrum disorder (ASD)-specific neuroanatomical variations from common variations in the general population, demonstrating that ASD-specific features were more closely associated with clinical symptoms, while shared features were more related to non-clinical attributes [17]. Therefore, we hypothesize that CVAE can similarly extract SCZ-specific features from fMRI data, facilitating more meaningful correlation analyses with symptoms and potentially providing deeper insights into the biological heterogeneity and pathological mechanisms of SCZ.

To reveal the disrupted spatiotemporal dynamic patterns of the brain in SCZ patients and untangle their heterogeneity, we attempt to address three progressively unfolding questions. Can the neurodynamic embedded CVAE model capture the disrupted spatiotemporal dynamic patterns of the brain in SCZ? Can the spatiotemporal dynamic abnormalities in SCZ be mapped to the PANSS symptom spectrum? What are the micro-scale mechanisms corresponding to multivariate correlation patterns of specific macro-scale spatiotemporal dynamic abnormalities and symptoms? To answer these questions, we designed a neurodynamic embedded CVAE model and optimized the model using our multi-site fMRI dataset, which includes 456 SCZ patients and 471 normal controls (NC). We first investigated whether SCZ-specific features exhibit different spatial and dynamic patterns compared to shared features, and whether they better capture symptom dimensions. Then, we used partial least squares (PLS) regression to find multivariate correlation modes between SCZ-specific features and PANSS scores. Finally, utilizing normalized gene expression data from the Allen Human Brain Atlas (AHBA), we further investigated different neurobiological correlates for each PLS correlation mode.

## Materials and methods

### Ethics approval and consent to participate

All methods were performed in accordance with the relevant guidelines and regulations. Written informed consent was obtained from all participants or their legal guardians. The study protocol was approved by the Ethics Committee of Peking University Sixth Hospital (approval number: 2010(52)). No identifiable information or images are included in the publication.

### Participants

Our multi-site dataset comprised participants recruited from six sites across five hospitals: Peking University Sixth Hospital (PKU6), Beijing Huilongguan Hospital (HLG), Xijing Hospital (XIAN), Henan Mental Hospital (XX_S and XX_G), and Renmin Hospital of Wuhan University (WUHAN). Detailed eligibility and exclusion criteria for participants and quality control measures for MRI data were consistent with those outlined in our previous studies [18, 19]. Standardized scanning protocols were implemented across all sites to ensure consistency. MRI data was acquired using either a 3 T Siemens Scanner (at PKU6, HLG, XIAN, and XX_S) or a 3 T GE scanner (at XX_G and WUHAN). At Henan Mental Hospital, where two scanners were utilized, participants were categorized into two distinct groups based on the scanner used, with no overlap between the groups. All individuals diagnosed with SCZ underwent comprehensive evaluations conducted by two qualified psychiatrists using the Structured Clinical Interview for DSM-IV axis I disorders (SCID-I/P, Patient Edition) to confirm their diagnosis. The final dataset included 927 subjects, consisting of 456 individuals with SCZ and 471 NC. All subjects met stringent criteria, including complete PANSS score information (for SCZ patients), high-quality MRI data that passed rigorous quality control procedures, and well-matched non-clinical characteristics such as gender, age, and education length. Table S1 provides an overview of participants’ clinical and non-clinical characteristics at each site.

### MRI acquisition and preprocessing

See Supplementary Methods for more details.

### Brain parcellations

See Supplementary Methods for more details.

### Neurodynamic embedded contrastive variational autoencoder (ND-CVAE) model

We adopted the general framework of CVAE [17, 20] to explore the application of SCZ-specific and SCZ/NC-shared neurodynamic autoencoders in SCZ and NC datasets. For a single regional sample, the model inputs include the fMRI time series ${\boldsymbol{y}}\in {{\mathbb{R}}}^{{n}_{t}}$ (length of time series: ${n}_{t}=225$), the network input ${\boldsymbol{u}}\in {{\mathbb{R}}}^{{n}_{t}}$ from other brain regions to this region weighted by SC, and the individual one-hot encoding ${\boldsymbol{c}}\in {{\mathbb{R}}}^{{n}_{{\rm{sub}}}}$ (total number of subject: ${n}_{{\rm{sub}}}=927$).

First, the SCZ-specific neurodynamic encoder and the SCZ/NC shared neurodynamic encoder share the same model structure. The former exclusively accepts input from SCZ samples, while the latter simultaneously accepts input from both SCZ and NC samples. For a single regional sample, the outputs of each encoder (also serves as input of neurodynamic source model) include the temporal states ${\boldsymbol{x}}\in {{\mathbb{R}}}^{{{n}_{s}n}_{t}}$ (where the number of states ${n}_{s}=2$), brain region-level parameters ${{\boldsymbol{\theta }}}^{{\rm{r}}}\in {{\mathbb{R}}}^{{m}_{r}}$ (with ${m}_{r}=2$ region-level parameters:), subject-level parameters ${{\boldsymbol{\theta }}}^{{\rm{s}}}\in {{\mathbb{R}}}^{{m}_{s}}$ (with ${m}_{s}=2$ subject-level parameters). Here, ${\boldsymbol{x}}$ and ${{\boldsymbol{\theta }}}^{{\rm{r}}}$ are extracted from the input (${\boldsymbol{y}}$, ${\boldsymbol{u}}$, ${\boldsymbol{c}}$) through a Long Short-Term Memory (LSTM) network with 32 hidden units, while ${{\boldsymbol{\theta }}}^{{\rm{s}}}$ is directly encoded by the subject’s one-hot encoding ${\boldsymbol{c}}$. Through these two autoencoders, we extract the SCZ-specific (only for SCZ samples) or shared temporal dynamics ${\boldsymbol{x}}$, brain region-level parameters ${{\boldsymbol{\theta }}}^{{\rm{r}}}$, and subject-level parameters ${{\boldsymbol{\theta }}}^{{\rm{s}}}$ from ${\boldsymbol{y}}$, ${\boldsymbol{u}}$, and ${\boldsymbol{c}}$ respectively.

Second, our neurodynamic model builds upon a previous network model of brain dynamics [12]. This model relaxes the constraints on the nonlinear differential equations governing brain region dynamics in the mean-field model (MFM) [8], which is derived from a simplified spiking neural mass model (NMM). Previous large-scale models often introduced parameters at the individual and brain region levels, such as the recurrent connection strength $W$ and global scaling factor $G$, to better fit observations and explain heterogeneity [8, 21]. Our model retains this concept, where brain region-level parameters (${{\boldsymbol{\theta }}}^{r}$) and subject-level parameters (${{\boldsymbol{\theta }}}^{s}$) can quantify the (dis)similarity between regions and provide insights into the heterogeneity of neural dynamics across brain regions and individuals. Therefore, we assume that the dynamics of a brain region are influenced not only by external inputs (such as subcortical nuclei) and inputs from other cortical brain regions but also by these parameters. We use a function (*f*) that can undergo deep-learning parameter optimization to characterize the changes in brain region dynamics. For a single sample from brain region *j*, the dynamics can be modeled as:

1 $${\dot{{\boldsymbol{x}}}}_{j}(t)=f[{{\boldsymbol{x}}}_{j}(t),{{\boldsymbol{\theta }}}_{j}^{{\rm{r}}},{{\boldsymbol{\theta }}}^{{\rm{s}}},{u}_{\text{ext}}(t),{u}_{j}(t)]+{\eta }_{j}(t)$$

2 $${u}_{j}(t)={\sum }_{i=1}^{n}{w}_{{ji}}{g}_{c}[{{\boldsymbol{x}}}_{j}(t)]$$

Here, ${{\boldsymbol{x}}}_{j}(t)\in {{\mathbb{R}}}^{{n}_{s}}$ represents the state of brain region *j* at time *t*. Large-scale neurodynamic models are derived from precise single-neuron spiking model, which undergo significant simplifications while retaining the form of state differential equations. As a result, the meaning of the state variables differs: for a single neuron, they are the membrane potential and ion channel conductances; for NMM, state variables represent mean firing rates, synaptic currents, and membrane potentials. In our whole-brain model, state variables refer to the generalized brain activity. ${{\boldsymbol{\theta }}}_{j}^{{\rm{r}}}$ and ${{\boldsymbol{\theta }}}^{{\rm{s}}}$ are the encoded parameters of brain region *j* from the encoders to characterize and capture the heterogeneity in brain region dynamics. ${u}_{\text{ext}}(t)$ represents external inputs shared by all brain regions of each subject. ${u}_{j}(t)$ represents the network input weighted by SC from other brain regions to brain region *j*. Samples were connected using group-averaged SC from the PKU6 site. In the control analysis, samples from the PKU6 site were connected using individual-level SC. The function *f* is specified as a two-layer fully connected network, with 32 hidden units and the rectified linear unit (ReLU) activation function. For ${{\boldsymbol{x}}}_{{aug}}=({{\boldsymbol{x}}}_{k},{{\boldsymbol{\theta }}}^{{\rm{r}}},{{\boldsymbol{\theta }}}^{{\rm{s}}},{u}_{\text{ext}},{u}_{j})$:

3 $${{\boldsymbol{x}}}_{k+1}=f({{\boldsymbol{x}}}_{{aug}})={W}_{2}\phi ({W}_{1}{{\boldsymbol{x}}}_{{aug}}+{b}_{1})+{b}_{2}$$

Where ${W}_{1}$ and ${W}_{2}$ are weights of fully connected network, ${b}_{1}$ and ${b}_{2}$ are biases, and ReLU activation function $\phi \left(x\right)=\max (0,{\boldsymbol{x}})$. For NC samples, their SCZ-specific states are zero-padded to ensure that the size of the ${\boldsymbol{x}}$ matrix for all samples input to the function *f* remains consistent.

Third, all NC and SCZ samples use the same state decoder. For NC samples, the input to the function *f* is used, i.e., the state matrix after zero-padding augmentation. The decoder is modeled as a linear transformation of the system states ${\boldsymbol{x}}(t)$ with Gaussian noise and no activation function:

4 $${\boldsymbol{y}}(t)=W\cdot {\boldsymbol{x}}(t)+b$$

### Evidence lower bound (ELBO)

In the classical VAE, the model’s loss function (−ELBO) can be cleverly decomposed into encoder and decoder losses. Given that our model incorporates a neurodynamic model, introducing neurodynamic constraints to the encoded temporal dynamics, the model’s loss can be further decomposed into an encoder loss without the prior distribution of temporal dynamics, a neurodynamic source model loss, and a decoder loss. For simplicity, we refer to the first term of the loss as the encoder loss, and only consider data from a single brain region sample, omitting the brain region index in the following analysis.

First, the encoder loss consists of SCZ-specific encoder loss and SCZ/NC shared encoder loss. The former calculates the loss only for SCZ samples in each batch, while the latter computes the loss for both SCZ and NC samples together in each batch. Since the model architectures and input structures for both autoencoders are the same, we can use ${L}_{\text{encoder}}$ to represent the encoder loss uniformly. The prior distributions of ${{\boldsymbol{x}}}_{0}$, ${{\boldsymbol{\theta }}}^{{\rm{r}}}$, ${{\boldsymbol{\theta }}}^{{\rm{s}}}$ are standard normal distributions $N(0,I)$. Here, ${{\boldsymbol{\theta }}}^{{\rm{s}}}$ is encoded by the subject’s one-hot encoding ${\boldsymbol{c}}$, while ${{\boldsymbol{x}}}_{0}$ and ${{\boldsymbol{\theta }}}^{{\rm{r}}}$ are extracted from the input (${\boldsymbol{y}}$, ${\boldsymbol{u}}$, ${\boldsymbol{c}}$) through LSTM network. Therefore,

5 $${L}_{{{\boldsymbol{\theta }}}^{{\rm{r}}}}=\text{KL}[q({{\boldsymbol{\theta }}}^{{\rm{r}}}\left|{\boldsymbol{y}},{\boldsymbol{u}},{\boldsymbol{c}}\right.){\rm{||}}N(0,I)]$$

6 $${L}_{{{\boldsymbol{x}}}_{0}}=\text{KL}[q({{\boldsymbol{x}}}_{0}\left|{\boldsymbol{y}},{\boldsymbol{u}},{\boldsymbol{c}}\right.){\rm{||}}N(0,I)]$$

7 $${L}_{{{\boldsymbol{\theta }}}^{{\rm{s}}}}=\frac{1}{n}\text{KL}[q({{\boldsymbol{x}}}_{0}\left|{\boldsymbol{c}}\right.){\rm{||}}N(0,I)]$$

8 $${L}_{\text{encoder}}={L}_{{{\boldsymbol{\theta }}}^{{\rm{s}}}}+{L}_{{{\boldsymbol{\theta }}}^{{\rm{r}}}}+{L}_{{{\boldsymbol{x}}}_{0}}$$

Second, all NC and SCZ samples share the same neurodynamic model, represented by the function *f* in Eq. (1), depicting the time evolution system of states. For NC samples, only the loss related to shared states is considered, and the loss function is defined as:

9 $${L}_{\text{source}}({\text{NCs}})=p({\boldsymbol{x}}\left|{{\boldsymbol{\theta }}}^{{\rm{r}}},{{\boldsymbol{\theta }}}^{{\rm{s}}},{u}_{\text{ext}}\right.,{u}_{j})={\prod }_{k=1}^{{n}_{t}}p({{\boldsymbol{x}}}_{k+1}({\text{shared}})\left|{{\boldsymbol{x}}}_{k},{{\boldsymbol{\theta }}}^{{\rm{r}}},{{\boldsymbol{\theta }}}^{{\rm{s}}},{u}_{\text{ext}},{u}_{j}\right.)$$

For SCZ samples, both the shared state and SCZ-specific state losses are considered simultaneously, and the loss function is defined as:

10 $${L}_{\text{source}}({\text{SCZs}})=p({\boldsymbol{x}}\left|{{\boldsymbol{\theta }}}^{{\rm{r}}},{{\boldsymbol{\theta }}}^{{\rm{s}}},{u}_{\text{ext}}\right.,{u}_{j})={\prod }_{k=1}^{{n}_{t}}p({{\boldsymbol{x}}}_{k+1}\left|{{\boldsymbol{x}}}_{k},{{\boldsymbol{\theta }}}^{{\rm{r}}},{{\boldsymbol{\theta }}}^{{\rm{s}}},{u}_{\text{ext}},{u}_{j}\right.)$$

Where ${{\boldsymbol{x}}}_{k+1}$ is defined in Eq. (3).

Third, all NC and SCZ samples use the same state decoder, which is modeled as a linear transformation of the system states with Gaussian noise. Expanding the likelihood over time, the decoder loss is expressed as:

11 $${L}_{\text{decoder}}=p(y\left|{\boldsymbol{x}},{{\boldsymbol{\theta }}}^{{\rm{r}}},{{\boldsymbol{\theta }}}^{{\rm{s}}},{u}_{\text{ext}}\right.)={\prod }_{k=1}^{{n}_{t}}N({y}_{k}\left|W\cdot {{\boldsymbol{x}}}_{k}+b\right.,{\sigma }_{o}^{2})$$

Where ***x*** includes both shared and SCZ-specific states. Finally, the overall loss function of the model is given by:

12 $${L}_{\text{dataset}}={L}_{\text{encoder}}+{L}_{\text{decoder}}+{L}_{\text{source}}(\text{NCs})+{L}_{\text{source}}(\text{SCZs})$$

### Model optimization

The optimization process of the model aims to minimize the total loss function in the Eq. (12). The trainable model parameters include the weights of the LSTM networks, the mean and variance ${M}_{1}$, ${M}_{2}$, ${V}_{1}$, ${V}_{2}$ for subject-level parameters ${{\boldsymbol{\theta }}}^{{\rm{s}}}$ in both encoders, parameters $W$ and $b$, ${\sigma }_{o}$ for the observation noise variance in the decoder, the weights of the state evolution function *f*, the state noise variance ${\sigma }_{s}$ in the source model.

The main training parameters were set as follows: learning rate was set to 0.003, batch size was 64 for randomly selecting subjects from both NC and SCZ groups, and the total number of training batches (*n*_batch_) was set to 30000. Adam optimizer was used for training the overall model. The reparameterization trick was employed for the gradient propagation of random variables. The fMRI time series for each of the 471 NC and 456 SCZ participants, comprising 100 brain regions each, were shuffled, and the inputs for each brain region were calculated to form the dataset (*n* = 92,700). Model optimization was performed using a five-fold cross-validation framework, with a random split of the data into training and test sets. Each fold consisted of a training set (80%, *n* = 74,160) and a test set (20%, *n* = 18,540). The loss function was optimized using only the training set while simultaneously calculating the model loss on both the training and test sets. The product-moment correlations of the encoded neurodynamic parameters across the cross-validation folds were calculated, and the fold with the highest average correlation coefficient with the other folds was selected as the optimal training model.

### Neuroimaging–clinic association analysis

We embarked on an exploration to investigate whether and how the shared and SCZ-specific parameters correlate with individual variations in nonclinical and clinical characteristics among SCZ participants. To accomplish this, we employed an approach inspired by representational similarity analysis (RSA) [22] to probe the individual variabilities in these encoding parameters. Subject dissimilarity was computed based on various demographic variables and symptoms. For numerical variables like age, education length and PANSS score, subject dissimilarity was quantified as the absolute Euclidean distance between their measurements, reflecting the difference in them. For categorical variables such as gender or scanning site, subject dissimilarity was set to 0 if the categorical variables matched and 1 otherwise.

Subsequently, we respectively computed the similarity of shared and SCZ-specific region-level and subject-level parameters between individuals, resulting in two dissimilarity matrices (456 subjects × 456 subjects). Taking the dissimilarity matrix for shared parameters as an example, the dissimilarity between individuals *i* and *j* was calculated using the Euclidean distance across all shared region-level and subject-level parameters (dimensionality=202). To ensure the robustness of RSA analysis, we performed 10 random samplings for each encoding parameter to calculate dissimilarity matrices. Then, we used unpaired-sample *t*-tests to determine whether the dissimilarity matrices related to demographic features and symptoms of participants were more strongly correlated with the dissimilarity matrices based on shared parameters or SCZ-specific parameters.

### Analysis of the inferred dynamical system characteristics

We employed the ‘hybr’ root-finding method from the *SciPy* toolkit to identify the fixed points of the system dynamics for all states of each subject node. Additionally, we utilized *TensorFlow*’s automatic differentiation to compute the Jacobian matrix and eigenvalues of the time evolution system for the states, allowing us to assess its stability. To better analyze the specificity between different brain regions, we set the network input to 0. For all states of each node, we performed 10 random samples from a uniform distribution in the range [−2, 2] to ensure robustness.

To assess the dynamic specificity of SCZ-specific states, we employed a deep learning classifier to evaluate the classification performance of fMRI signals generated with or without SCZ-specific state information. The deep learning classifier consists of two modules: a spatial domain encoding module and a temporal domain encoding module. The spatial domain encoding module utilizes two cascaded residual network (ResNet) modules, while the temporal encoding module employs three cascaded Transformer modules. The final Transformer module is followed by a fully connected layer with a sigmoid activation function, responsible for predicting whether the sample belongs to SCZ or NC. The entire model was trained using the Adam optimizer, with key training parameters set as follows: an initial learning rate of 3 × e^−4^, decreasing by a factor of 10 every 10 epochs; a batch size of 64; and a total of 20 epochs. Initially, we evaluated the model’s classification performance on our large-scale SCZ dataset, randomly splitting the dataset into a training set (70%) and a test set (30%). Subsequently, within the previously trained neurodynamic source model, for each SCZ individual, we separately generated 100 simulated time series by adding either shared parameters only or both shared parameters and SCZ-specific parameters. We then tested and compared the classification performance using the trained model on these simulated time series.

### PLS between SCZ-specific features and behavioral symptoms

Considering that SCZ-specific features capture more SCZ-specific information, we employed PLS regression to further explore their association with the clinical symptom spectrum. Given the high dimensionality of SCZ-specific states, we used the *Highly Comparative Time-Series Analysis* (HCTSA, v1.06) toolbox [23] for extensive time-series feature extraction and analysis of complex temporal dynamics. Previous literature [24] has confirmed 44 robust time-series features based on fMRI data, covering a wide range of temporal dynamic characteristics, including linearity, non-linearity, non-stationarity, and stochasticity, and showing relevance with cognition and behavior. Although these 44 time-series features provide a comprehensive and quantitative description of the extracted SCZ-specific states, there is still a high degree of intercorrelation within the features. To reduce the redundancy of PLS input features, we further tested the intercorrelations of these 44 features in SCZ-specific states. Features with absolute values of the intercorrelation coefficients exceeding 0.5 were considered as feature clusters, and the representative feature with the highest sum of the absolute values of the intercorrelation coefficients was selected as the representative feature for each cluster. Based on previous research [24], the selected 9 time-series features can be further classified into 4 dimensions according to their associated keywords, including distribution, predictability, non-stationarity, stochasticity.

Specifically, distribution features encompass ‘*mean*’, ‘*standard deviation*’, and ‘*FitKernelSmoothraw.entropy*’. ‘*mean*’ and ‘*standard deviation*’ are the mean and standard deviation of the input time-series respectively. ‘*FitKernelSmoothraw.entropy*’ represents the entropy for a kernel-smoothed distribution of the input time-series. Predictability features encompass ‘*trev.3.denom*’ and ‘*StdNthDer.4*’. ‘*trev.3.denom*’ calculates normalized nonlinear autocorrelation of the input time-series. ‘*StdNthDer.4*’ computes the standard deviation of the fourth derivative of the input time-series. Non-stationarity features encompass ‘*PPtest.meanstat*’, ‘*Walker.distdiff*’, and ‘*VisibilityGraph.norm.expnlogL*’. ‘*PPtest.meanstat*’ carries out the Phillips-Perron (PP) unit root test to ascertain whether a unit root exists within the input time-series. ‘*Walker.distdiff*’ describes to what extent the original time series can be represented by the trajectory of a walker adheres to specific kinematic rules. ‘*VisibilityGraph.norm.expnlogL*’ corresponds to the likelihood of the degree distribution under an exponential-fitted distribution in a visibility graph constructed based on ‘normal’ method. Stochasticity features encompass ‘*VarRatioTest.4.1.stat*’, executing variance ratio test to test whether the input time-series exemplifies a random walk.

To compare the dynamic differences between brain regions, we calculated dynamic FC matrices for each subject, each state, and each of the 52 time windows (with a window length of 40 seconds and a step size of 8 s), resulting in a 100 × 100 × 52 matrix for each subject. Based on this, we computed the dynamic correlation variability of each brain region’s state with the states of other brain regions within the same subject, serving as the 10th dynamic feature of the temporal state:

13 $${V}_{k}=1-\bar{\text{corrcoef}}({F}_{i,k},{F}_{j,k})\left(i,j=1,2,3,...,N,i\ne j\right)$$

Where *k* represents the brain region number, *i* and *j* represent the time window numbers, *N* = 52 is the total number of time windows, *F*_*i,k*_ and *F*_*j,k*_ (*n* = 99) represent linear correlation vectors of the *k* brain region’s state with the states of all other brain regions for different time windows.

We extracted the aforementioned 10 dynamic features for each brain region from each subject (456 subjects × 100 regions × 2 SCZ-specific states × 10 dynamic features). The two region-level SCZ-specific parameters ${{\boldsymbol{\theta }}}^{{\rm{r}}}$ (456 subjects × 100 regions × 2 parameters) and two subject-level SCZ-specific parameters ${{\boldsymbol{\theta }}}^{{\rm{s}}}$ (456 subjects × 2 parameters) were concatenated, resulting in a reshaped SCZ-specific features matrix of size 456 × 2202. This matrix served as the predictor variables in the PLS regression. Subject-level scores for PANSS negative, positive, and general scores (456 subjects × 3 scores) were taken as response variables in the PLS. The age, gender, and site were regressed out from the PANSS scores. The core functionality of PLS regression is to optimize weighted combination patterns to obtain pairwise latent components (LC), one for the PANSS profiles and the other for SCZ-specific encoded features, with maximal covariance. After identifying the LCs with PLS, we calculated the *P* values for the LCs using a permutation test (randomly shuffling the order of PANSS scores among SCZs, 1,000 permutations) to identify significant LCs after correction for multiple comparisons (*P*_FDR_ < 0.05). For each significant LC, we calculated loadings for the three PANSS subscale scores by measuring Pearson correlations between the three subscale scores and the corresponding latent variable driven by the PANSS scores. Using a similar procedure, we obtained SCZ-specific parameter loadings for each significant LC. We averaged SCZ-specific parameter loadings at both the brain region and dynamic feature levels, resulting in brain loading maps and feature loadings for the predictor variables.

### NeuroSynth term-based meta-analysis

See Supplementary Methods for more details.

### Neuroimaging–transcription association analysis

To provide further neurobiological underpinnings of the two brain loading maps, we explored the potential mechanisms underpinning the two neurodynamic-clinic association patterns using the AHBA [25] transcriptome dataset. This dataset comprised regional microarray expression data from the postmortem brains of six donors (five men and one woman, ages ranging from 24 to 57). We employed the *Abagen* toolbox [26] to preprocess the gene expression data and map them to the 100 cortical parcellations in MNI space. Following *Abagen*’s recommended protocol, our standardization preprocessing pipeline consisted of probe reannotation, data filtering, probe selection, sample selection, assignment to brain regions, normalization of gene expression values, and averaging across regions (see Supplementary Methods for more details). In the end, this workflow resulted in a regional gene expression matrix with 100 rows (representing brain regions) and 15,633 columns (representing the remaining genes).

Following that, PLS regression was again and respectively applied to evaluate the relationships between gene expression and the two brain loading maps, with gene expression serving as predictor variables in the PLS and the latter as response variables. We employed Brain Surrogate Maps with Autocorrelated Spatial Heterogeneity (BrainSMASH) [27] to generate the 1000 surrogate maps while rigorously preserving Spatial Autocorrelation (SA) for the two brain loading maps. Then we calculated the *P* values of permutation test for the pairwise LC using the generated 1000 surrogate maps to identify whether the explained variance in the components was significantly larger than that achieved by chance. Furthermore, we performed bootstrapping (1000 times) to estimate the error of the weight of each gene, and the normalized weight of each gene was generated by dividing the weight by the estimated error. Finally, to further elucidate their roles, we performed a gene ontology (GO) biological process enrichment analysis for statistically significant genes using the ToppGene portal [28]. To validate the enrichment results mentioned above, we selected the top and bottom 1500 ( ~ 10%) ranked genes most correlated with the two brain loading maps as a validation gene set. To address multiple comparisons, we applied the BH method for FDR (FDR-BH correction) (*P* < 0.05) during the analysis.

## Results

### Model optimization and evaluation

**Fig. 1:**
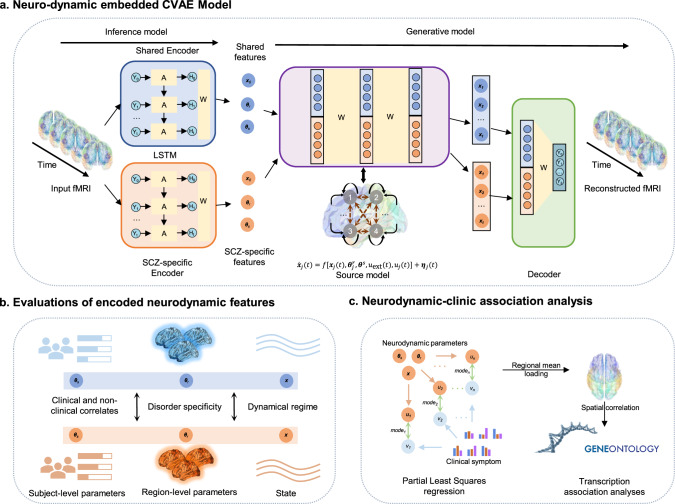
Overview of the study scheme. **a** Neurodynamic embedded contrastive variational autoencoder (ND-CVAE) Model. Shared and SCZ-specific features were extracted from two encoders and used to construct the source model of brain dynamics. **b** Evaluations and comparisons of shared and SCZ-specific features, including subject-level parameters ${{\boldsymbol{\theta }}}^{{\rm{s}}}$, region-level parameters ${{\boldsymbol{\theta }}}^{{\rm{r}}}$, and the hidden states of node systems ${\boldsymbol{x}}$. **c** Neurodynamic-clinic association analysis. The relationship between the encoded features and participant properties were mapped via PLS regression. The underlying potential micro-transcriptomic mechanisms were explored by transcription association analyses.

The foundational model and workflow of this study are illustrated in Fig. 1. We integrated a neurodynamic model into the classical CVAE framework, specifically extracting shared and SCZ-specific features from respective encoders (Fig. 1a). These features contribute to the construction of the brain dynamics source model. The constraints imposed on the neurodynamic model confer biophysical significance to shared and SCZ-specific features, encompassing subject-level parameters (${{\boldsymbol{\theta }}}^{{\rm{s}}}$), region-level parameters (${{\boldsymbol{\theta }}}^{{\rm{r}}}$), and the hidden states of node systems (${\boldsymbol{x}}$). Subsequently, we examined the characteristics of these parameters, correlated them with symptoms, and explored their underlying micro-transcriptomic mechanisms (Fig. 1b, c).

Under the five-fold cross-validation framework, the losses of the training and testing sets across all five folds were synchronized and consistently reduced (Figure S1a). Specifically, the total loss for the encoder, source model, and decoder stabilized after approximately 5000, 10,000, and 25,000 training batches, respectively (Figure S2). Encoded parameters exhibited relative stability across the cross-validation folds, with product-moment correlations of across the cross-validation folds showing mean ± SD of 0.76 ± 0.05 for ${{\boldsymbol{\theta }}}^{{\rm{r}}}$, and 0.59 ± 0.18 for ${{\boldsymbol{\theta }}}^{{\rm{s}}}$ (Figure S1c, d). Together, these findings suggest that the model exhibits a good fit within our dataset. We selected the most stable fold, identified as the one with the highest average correlation coefficient of encoded parameters with the other folds, as the optimal training model.

Furthermore, when utilizing a different atlas (Desikan-Killiany atlas with 68 regions), we observed that the model optimization process and encoded parameters remained robust to changes in brain parcellations (Figure S3). We then conducted a control analysis using individual-level SC for samples at the PKU6 site (Figure S4). The subject mean correlations between the external inputs (${u}_{\text{ext}}$) received by each brain region using individual-level and group-average SC were high (mean ± SD of 0.86 ± 0.05), with no significant between-group difference (*P* = 0.69). The results obtained using individual-level SC, which closely mirror those at the group level (i.e., stable model optimization and meaningful encoding parameters), indicate that brain dynamics were not significantly impacted by minor variations in SC. This finding aligns with previous research, demonstrating that changes in structural connections are not directly linked to dynamic metrics (FC) measured in patients [29], while changes in dynamic parameters (such as global coupling strength, E/I ratio) can shift the dynamics from the optimal healthy regime and account for the dysfunction observed in FC [10, 29–31].

Subsequently, we tested the effect of parameter space dimensionality on the model’s performance and stability (Figure S5). Given the instability and reduced generalization ability introduced by the third parameter, we will focus on results based on a parameter space dimensionality of 2 in our subsequent study.

### Evaluations of SCZ-specific subject- and region-level parameters

**Fig. 2:**
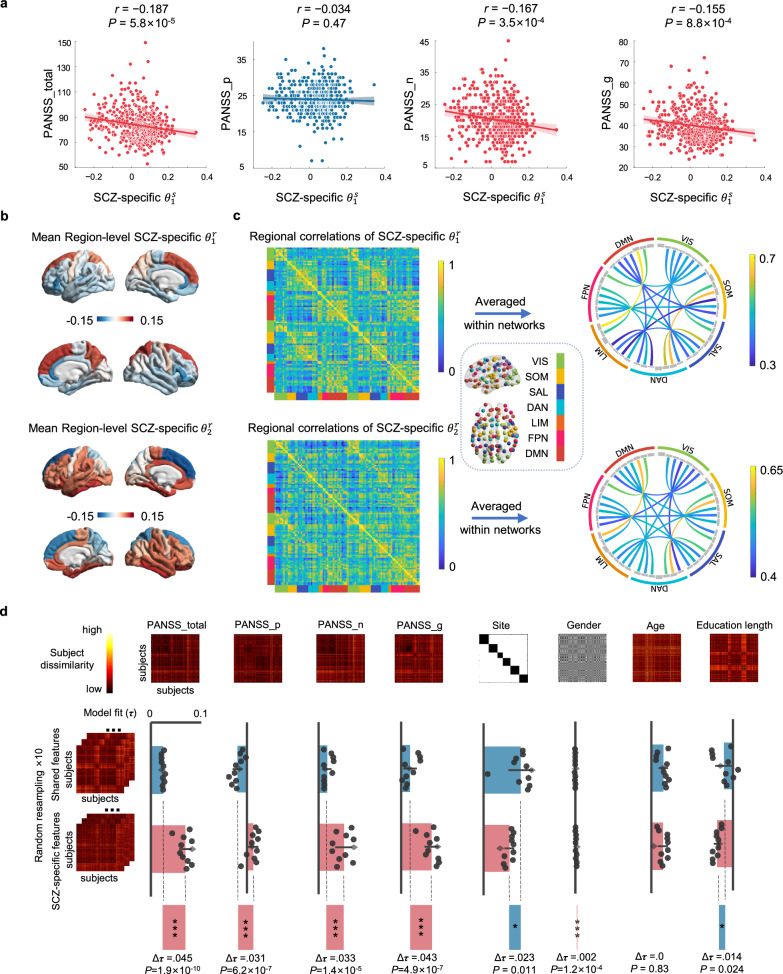
Evaluations of subject-level parameters and region-level parameters. **a** Scatter plots showing the associations between SCZ-specific ${{\boldsymbol{\theta }}}_{1}^{{\rm{s}}}$ and PANSS total, positive, negative and general scores. **b** Regional mean SCZ-specific ${{\boldsymbol{\theta }}}_{1}^{{\rm{r}}}$ and ${{\boldsymbol{\theta }}}_{2}^{{\rm{r}}}$ (100 regions) inferred by averaging the parameter matrix (456 subjects × 100 regions) on the subject dimension. **c** Regional correlations of SCZ-specific ${{\boldsymbol{\theta }}}_{1}^{{\rm{r}}}$ and ${{\boldsymbol{\theta }}}_{2}^{{\rm{r}}}$ (100 regions × 100 regions) inferred by taking the Pearson correlation coefficients of the parameter matrix (456 subjects × 100 regions) on the subject dimension. **d** Dissimilarity matrices of shared and SCZ-specific subject-level parameters and region-level parameters compared with dissimilarity based on different participant properties. Model fit was assessed using the Kendall rank correlation coefficient (Kendall *τ*). Black circles represent the results of 10 random resampling. Gray diamonds represent the results of directly selecting the mean value.

First, we directly conducted Pearson correlation analysis to examine the correlations between the subject-level parameters extracted by the model and the PANSS total and subscale scores in SCZ. As shown in Fig. 2a, we observed significant correlations of SCZ-specific subject-level parameters with PANSS total score (*r* = −0.187, *P* = 5.8 × 10^-5^), negative subscale score (*r* = −0.167, *P* = 3.5 × 10^-4^), and general subscale score (*r* = −0.155, *P* = 8.8 × 10^-4^). These correlations achieved significance across all 5/5 cross-validation folds (Fig. S1b), demonstrating robustness to different brain parcellations (Fig. S3c), individual-level SC (Fig. S4d), and after regressing out various covariates (Table S2).

We averaged the shared and SCZ-specific region-level parameter matrices along the individual dimension to obtain brain maps for the four parameters. It can be observed that all four parameters exhibit certain gradient variations across the brain. Meanwhile, the two SCZ-specific region-level parameters present distinct features (Fig. 2b), while the two shared region-level parameters exhibit similar characteristics (Fig. S6). Subsequently, regional correlations of SCZ-specific ${{\boldsymbol{\theta }}}_{1}^{{\rm{r}}}$ and ${{\boldsymbol{\theta }}}_{2}^{{\rm{r}}}$ (100 regions × 100 regions) were inferred by calculating the Pearson correlation coefficients of the parameter matrix (456 subjects × 100 regions) along the subject dimension (Fig. 2c) to examine the similarity of region-level parameters across brain regions. High similarity indicates consistent variations between two brain regions across different individuals, and vice versa. Notably, the first SCZ-specific region-level parameter shows low similarity between the DMN and SOM\SAL\DAN networks, while the second SCZ-specific region-level parameter exhibits low similarity between the VIS and DMN\FPN\DAN networks.

We subsequently explored the associations between features of individuals with SCZ inferred from two encoders and their clinical symptoms, as well as other potential confounding variables such as site, age, sex, and education length. In this exploration, we utilized the representational similarity analysis (RSA) method [22]. Specifically, we calculated a dissimilarity matrix (456×456) for SCZ-specific features, shared features, PANSS total score, three PANSS subscale scores, as well as age, sex, and site for individuals with SCZ, based on the Euclidean distance (see Methods). For the two types of encoded (shared and SCZ-specific) features, we repeated the sampling and dissimilarity matrix calculations 10 times to ensure robustness. Finally, we separately computed the Kendall rank correlation (Kendall *τ*) between the lower triangles of the dissimilarity matrices for the two types of encoded features and the dissimilarity matrices related to clinical and demographic characteristics. The results indicated that the subject dissimilarity matrix obtained from SCZ-specific features exhibited significantly higher correlations with symptoms (PANSS scores) compared to the shared features (*P* < 0.05). Conversely, the shared features predominantly captured two non-clinical variations: education length and site (Fig. 2d). This underscores the differential effectiveness of shared and SCZ-specific encoders in extracting symptom dimensions from common properties.

### Evaluations of SCZ-specific states

**Fig. 3:**
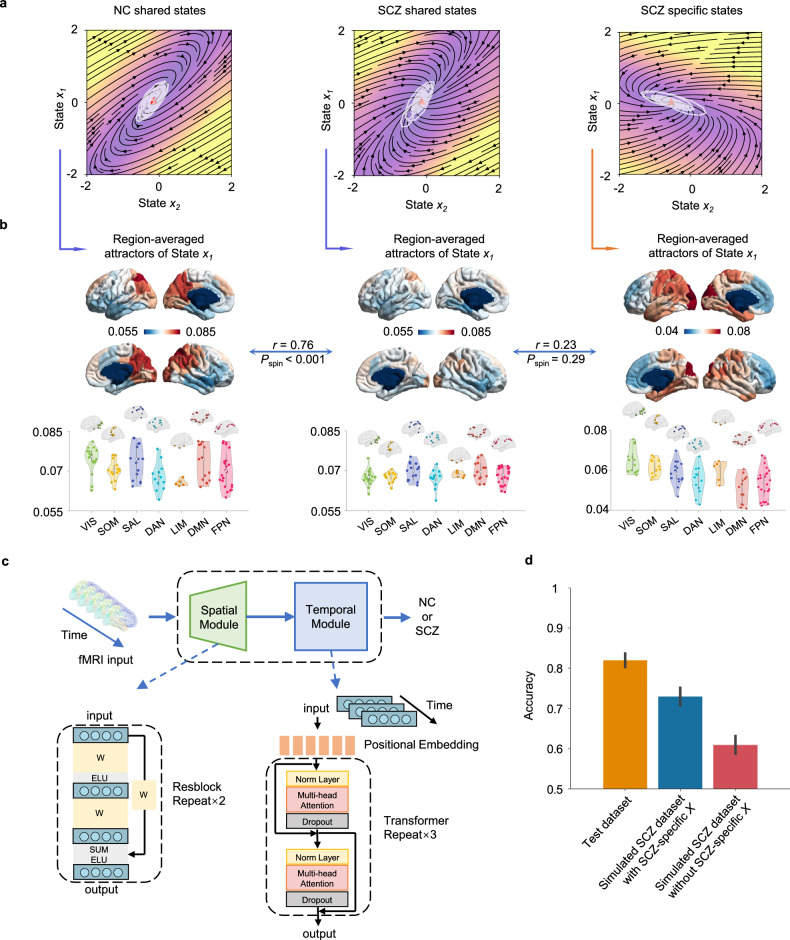
Evaluations of the hidden states of node systems. **a** Inferred dynamics in state space of two example nodes. The vector field is assessed assuming zero network input and the inferred parameters. Background color indicates velocity magnitude; white lines indicate encoded time series of the node states; red triangles indicate fixed points. **b** Regional averaged attractors of state ***x***_1_ (100 regions) inferred by averaging the attractor matrix of state ***x***_1_ (471 NCs or 456 SCZs ×100 regions) on the subject dimension. From left to right are the shared state *x*_1_ of the NC group, the shared state ***x***_1_ of the SCZ group, and the SCZ-specific state ***x***_1_ of the SCZ group. The colorbar range is set identically for the first two groups. The shared states’ attractors of the NC group and the SCZ group exhibit similar brain gradient patterns, with the latter showing smaller inter-regional variances. However, the specific state ***x***_1_ of the SCZ group present distinctive gradient patterns compared to both shared states. **c** Spatiotemporal classification model based on deep-learning. The spatial module incorporates fully connected layers (ResNet) to process spatial information at each time step, while the temporal module utilizes attention layers (Transformer) to model temporal brain source dynamics. **d** The classification accuracy of the spatiotemporal model on different datasets. From left to right, the three columns represent the classification accuracy of the model on three different test sets: the original test set in the training, and simulated test sets generated with and without SCZ-specific states. Compared to the simulated test set generated without SCZ-specific states, the simulated test set using SCZ-specific states exhibits higher classification accuracy. The result suggests that SCZ-specific states contribute to capturing information more accurately from SCZ brain activity.

After evaluating SCZ-specific subject- and region-level parameters of the SCZ, we further evaluated the temporal features extracted by the model, namely the time-varying state. We first examined the attractors of the neurodynamic model for all brain regions. Using the root-finding method, we calculated the fixed points and eigenvalues of the neurodynamic systems for all brain regions in the absence of external input. In all cases, the eigenvalues of the systems at these fixed points were real and negative, indicating stability. As shown in Fig. 3a, for examples of one brain region from a control individual (left) and one brain region from an individual with SCZ (center and right), all the extracted states (white lines) exhibited oscillations around the fixed points. Among all samples, only one brain region from one control individual showed bistability, indicating two fixed points (Fig. S7a). However, the system remained stable. Subsequently, we averaged the attractors for the first state across individuals for each group, and the brain maps for average attractors are shown in Fig. 3b. The NC and SCZ groups exhibited similar hierarchical structures in the fixed points for shared states (*r* = 0.76, *P*_spin_ < 0.001), with the difference being lower intra-network variance in the SCZ group. However, the fixed points for SCZ-specific states in the SCZ group did not display similar cortical patterns to the shared states (*r* = 0.23, *P*_spin_ = 0.29). We employed BrainSMASH [27] to generate the 1,000 surrogate maps for the above spin test. Similar conclusions were drawn for the attractors of the second state across the three groups (Fig. S7b). This provides evidence for the specificity of the attractors on SCZ-specific states.

Subsequently, we employed a deep learning model to further validate whether SCZ-specific states could capture information about SCZ-specific brain activity. The model structure consists of a spatial module and a temporal module connected in series (Fig. 3c). The spatial module incorporates fully connected layers to process spatial information at each time step, while the temporal module utilizes attention layers to model temporal brain source dynamics. We first randomly split the real dataset into training (70%) and testing (30%) datasets and used random sampling within the encoded state space to generate two simulation testing datasets: one generated with only shared states and the other generated with both shared and SCZ-specific states. We trained this model on real training dataset and tested the classification accuracy on real and simulation testing datasets. We repeated above steps 10 times to ensure robustness. As shown in Fig. 3d, for real testing dataset, the classification accuracy was: mean = 82.1%, SD = 2.3%. For simulation testing datasets with SCZ-specific states, the classification accuracy was: mean = 73.3%, SD = 2.8%. In contrast, when excluding SCZ-specific states for generation, the classification accuracy significantly decreased to: mean = 61.4%, SD = 3.1%. These results demonstrate that SCZ-specific states can capture more disorder-specific information from SCZ brain activity.

### Association between SCZ-specific features and behavioral symptoms

Subsequently, we utilized PLS regression to map the relationship between the SCZ-specific parameters and states obtained from the constructed model and their association with symptoms. The goal was to elucidate how SCZ-specific parameters and states contribute to the generation of a complex spectrum of symptoms. Considering the high dimensionality of SCZ-specific encoded states, we employed the HCTSA toolbox [23] for extensive time-series feature extraction and analysis of complex temporal dynamics. A previous literature [24] has confirmed 44 robust time-series features for fMRI data, covering a wide range of temporal dynamic characteristics. Considering the redundancy of these 44 features, we further selected 9 representative features based on the intercorrelations of these 44 features (Fig. S8) to provide a comprehensive and quantitative description of the extracted SCZ-specific states. To compare the dynamic differences between brain regions, we calculated the dynamic correlation variability of each region’s state with others (see Methods). Finally, we extracted the mentioned 10 dynamic metrics for each temporal state, forming a 456 × 2202 feature matrix by concatenating SCZ-specific subject-level and region-level parameters as neuroimaging predictor variables. This matrix was then correlated with the three PANSS subscale scores in PLS regression analysis to explore their multivariate relationships.

**Fig. 4:**
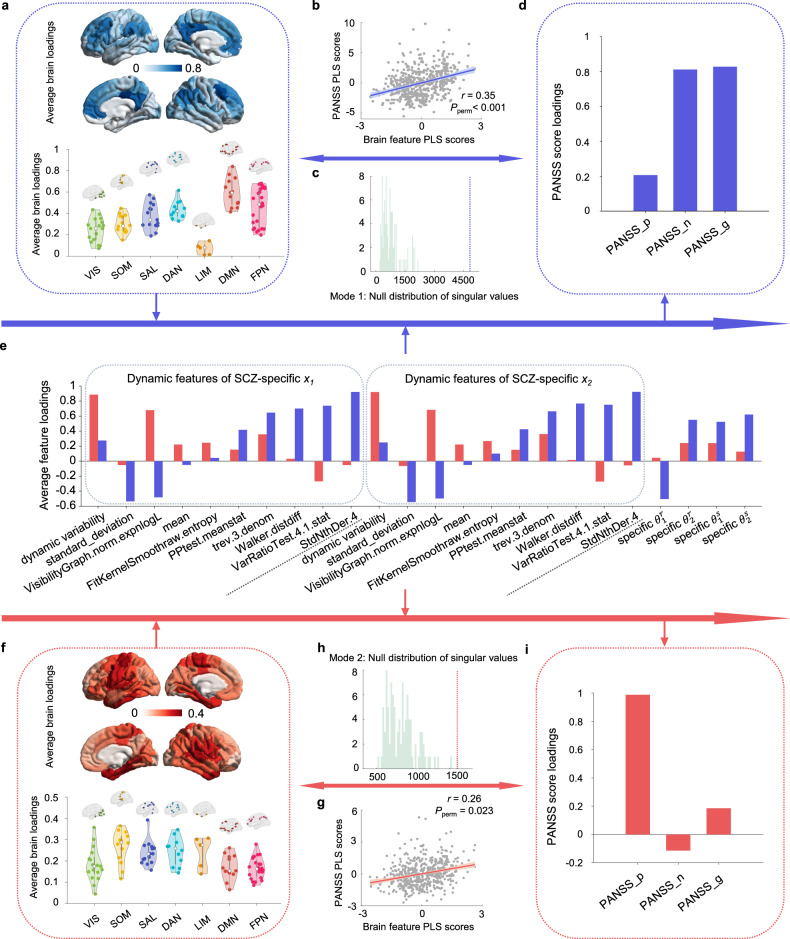
Spatiotemporal dynamic patterns in two PLS modes. **a**, **f** Average brain loadings obtained by averaging the loadings of brain features along the feature dimension in the two PLS modes. **b**, **g** Scatter plots of brain feature PLS scores and PANSS PLS score, derived from linear combinations of brain spatiotemporal dynamic features and PANSS scores, respectively. **c**, **h** Bar plots depicting the null distribution of singular values of the covariance matrices through permutation testing, with a red dashed line marking the actual value. **d**, **i** PANSS score loadings calculated by measuring Pearson correlations between the three PANSS scores and the corresponding latent variable (i.e., PANSS PLS scores) driven by the PANSS scores **e** The average feature loadings obtained by averaging the loadings of brain features along the brain dimension in the two PLS modes. The blue bars represent mode 1, and the red bars represent mode 2.

The results reveal two significantly correlated modes between imaging and symptoms (Fig. 4): mode 1 (*r* = 0.35, *P*_perm_ < 0.001) and mode 2 (*r* = 0.26, *P*_perm_ = 0.023). In mode 1, DMN, FPN, and DAN networks show higher loadings, while LIM exhibits lower loadings (Fig. 4a). Meanwhile, PANSS negative and general subscales are predominant (Fig. 4d). In terms of imaging features, SCZ-specific subject-level and region-level parameters, together with predictability- and stochasticity-type dynamic features such as ‘*trev.3.denom*’, ‘*StdNthDer.4*’, and ‘*VarRatioTest.4.1.stat*’ play a major role (Fig. 4e). In mode 2, the PANSS symptom subscales are primarily dominated by positive scores (Fig. 4i). Regarding imaging features, dynamic variability and ‘*VisibilityGraph.norm.expnlogL*’ take precedence (Fig. 4e). On the regional level, slightly higher loadings are observed in SOM and SAL (Fig. 4f).

### Cognitive correlates of two PLS modes

Cognitive impairment is a significant feature of SCZ, affecting various cognitive domains such as attention, memory, reasoning, and processing speed [32]. To empirically explore the relationship between the two PLS modes and different cognitive domains, we performed a term-driven meta-analysis with NeuroSynth [33], a frequently utilized tool for delineating extensive brain patterns in the context of their cognitive significance. We utilized 24 classic behavior- and cognition-related topic terms according to previous studies [34], covering a wide range of visual, motor, and cognitive functions. For each PLS mode, we examined cognitive topics corresponding to the average brain loadings (Fig. S9). Both modes showed robust associations with motor and multisensory processing, as well as action, consistent with known cognitive impairments in SCZ [35]. Additionally, mode 2 demonstrated an additional influence on pain and auditory processing, which seems to be related to auditory hallucinations.

### Transcriptomic association analysis for two PLS modes

**Fig. 5:**
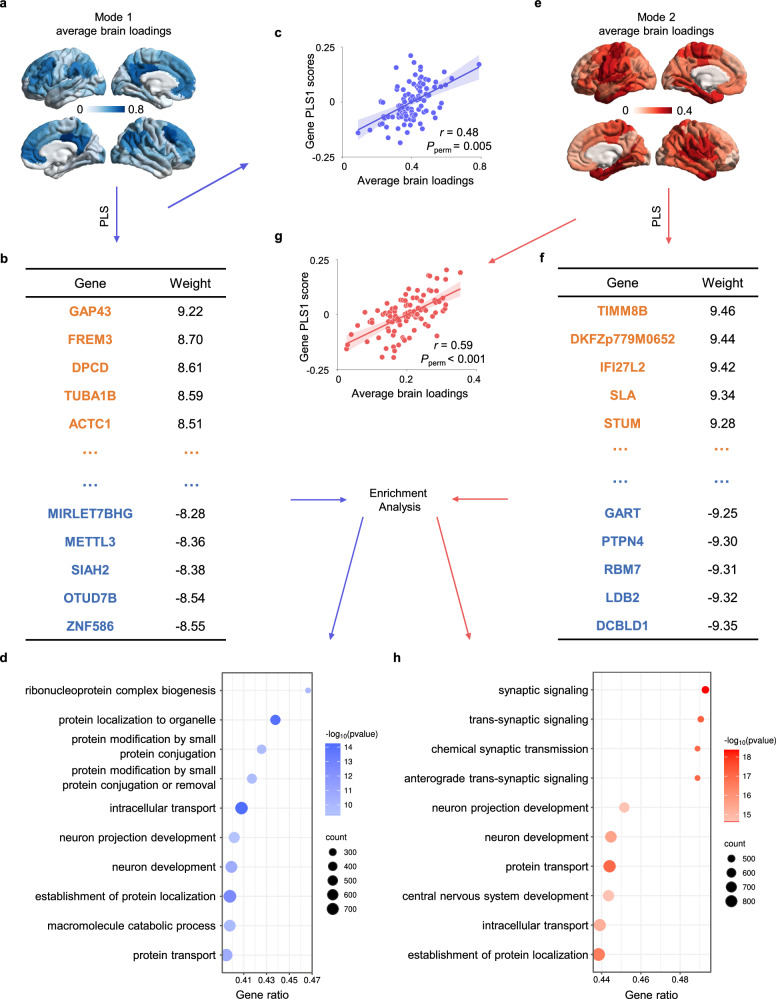
Micro-transcriptomic mechanisms of brain loading pattern in mode 1 and mode2. **a**, **e** Average brain loadings in mode 1 and mode 2. **b**, **f** Genes that positively and negatively weighted values for the subsequent GO biological process enrichment. **c**, **g** Scatter plots of Gene PLS scores and average brain loadings in mode 1 and mode 2. **d**, **h** The first 10 biological process terms of enrichment analysis for genes with weight |z | > 3. The size of the circle represents the number of genes involved in the specific term, and the color represents the corrected *P* values. (*P* < 0.05, FDR-BH corrected).

Lastly, we conducted transcriptomic association analysis and GO enrichment analysis to gain insights into the neurobiological underpinnings of the two PLS modes. We first mapped normative regional gene expression profiles from the AHBA [25] to the 100 functional parcellations, generating a gene expression matrix (100×15,633). Subsequently, PLS regression analysis between average brain loadings in each mode (response variables, Fig. 5a, e) and gene expression (predictor variables) was performed to identify the potential gene-level mechanism. Both latent genetic components of average brain loadings of mode 1 (*r* = 0.48, *P*_perm_ = 0.005) and mode 2 (*r* = 0.59, *P*_perm_ < 0.001) achieved significance in permutation tests of brain rotation. By estimating the normalized weights (Z-score) for each gene through bootstrap resampling 1,000 times, the resulting 3685 (mode1) and 3257 (mode2) subsets of positively and negatively weighted genes (|z | > 3) were enriched for the GO biological process (Fig. 5b, f).

The enrichment results are shown in Fig. 5d, h. Both modes share common items related to fundamental neuron development and protein transport, including neuron development (GO:0048666), neuron projection development (GO:0031175), protein transport (GO:0015031), intracellular transport (GO:0046907), and establishment of protein localization (GO:004518). Mode 1 is specifically enriched in cellular processes such as ribonucleoprotein complex biogenesis (GO:0022613) and macromolecule catabolic processes (GO:0009057). In contrast, mode 2 is more associated with signaling transduction and nervous system development, such as synaptic signaling (GO:0099536), trans-synaptic signaling (GO:0099537), chemical synaptic transmission (GO:0007268), and central nervous system development (GO:0007417). Subsequently, we calculated Pearson correlations between the gene expression matrix and average brain loadings, and selected the top and bottom 1500 genes (approximately 10%) for each mode for validation, yielding similar conclusions (Fig. S10).

## Discussion

This study employed an innovative approach by integrating neurodynamic models into the classical CVAE framework, aiming to extract SCZ-specific brain activity features from neuroimage data and elucidate the intricate relationships spanning microscale molecular mechanisms, large-scale neural circuits, and individual-level symptoms and cognition in SCZ. Our findings reveal robust disruptions in the spatiotemporal dynamic patterns of the brain in SCZ. Furthermore, we identify two sets of multivariate correlation modes between SCZ-specific spatiotemporal dynamic features and symptom profiles, which illuminate individual variations in SCZ and suggest distinct genetic, molecular, and neurodynamic mechanisms.

### Evaluations of SCZ-specific features

The first major finding of our study is that the method of integrating neurodynamic models into the CVAE framework fits well in our multisite dataset. This good fit is reflected in two aspects. Firstly, we observed a stable and synchronized decrease in training and testing dataset loss. Secondly, the extracted SCZ-specific features showed significant correlations with PANSS scores, particularly in the domains of negative and general symptoms. Our results advance the application of the CVAE framework in two steps [17]: first achieving application on the high-dimensional 4D fMRI time-series data, and second imparting biological significance to the features extracted by the encoder through the constraints of neurodynamic models. This enhances the interpretability of encoded parameters and enables individual-level neurodynamic studies in SCZ.

The observed distinctive cortical gradient in the brain maps of region-level parameters and attractors obtained from neurodynamic systems provide spatial insights into the heterogeneity of brain regions in SCZ and a deeper understanding of neurodynamic related to SCZ. It is noteworthy that all shared and SCZ-specific region-level parameters exhibit higher similarity within networks than between networks. This not only further suggests a better fit of the model but also indicates that the captured brain region parameters reflect homogeneity within brain regions, such as FC and redundant effects in information interaction, rather than heterogeneity, such as synergistic effects in information interaction [36]. Contrary to intuition, our results show that the majority of brain regions exhibit monostable regime, which can be described as noisy fluctuations around a single stable fixed point (attractor) at the node level. Although this is similar to the results in the original neurodynamic model [12], it seems to deviate from our expectations. Specifically, a signature of nonlinear brain dynamics is multistability, which refers to the coexistence of multiple stable brain activity patterns [37], and external influences or noisy dynamics can drive transitions between these brain states [38]. However, since our neurodynamic model does not assume multiple stability in its architecture and the optimization goal of the model is to capture observed neural activity in all aspects of time and space, a multiple attractor pattern of the system was not found. Together, we believe that there is still a need for further improvement in the structure and optimization of this neurodynamic model to achieve a stronger understanding of the nature of brain dynamics.

The results of representational similarity analysis and the decrease in classification accuracy observed in deep learning models when excluding SCZ-specific states further emphasize the unique contribution of SCZ-specific features in capturing symptom dimensions. The results of the deep learning model also demonstrate that the high classification performance on SCZ versus NC may be related to capturing unique information about SCZ brain activity. This further suggests the possibility of pre-training deep classification models [39] with simulated fMRI carrying SCZ-specific dynamic information to address the deficiency in the SCZ sample set and may contribute to the interpretability of deep learning models. It is noteworthy that, following the application of CVAE as described in the original work [17], we did not incorporate the loss term enforcing the SCZ-specific features of the NC group to be zero in the loss function. Consequently, the SCZ-specific features for the NC group samples in the model were random and meaningless. Therefore, we demonstrated the importance of SCZ-specific neurodynamic parameters by evaluating the classification performance of SCZ samples carrying different SCZ-specific dynamic information.

### Mapping the correlation patterns between SCZ-specific features and symptoms

Our second key finding involved using PLS regression to identify multivariate correlations between two sets of neurodynamic features and symptoms, thereby further enhancing our multidimensional understanding of the complex relationships between SCZ-specific parameters, states, and behavioral symptoms in SCZ. This approach is more reasonable than using a subtype-based method to explain the heterogeneity of SCZ because multiple symptom domains are continuous variations observed at varying degrees across patients [40], leading to high variance even within subtypes [41]. Hence, considering that multiple pathophysiological processes may underlie individual differences in SCZ and that SCZ inter-individual variability may reflect different degrees of expression of these pathophysiological mechanisms, it is intriguing to investigate multidimensional pathological mechanisms coexisting in individuals with SCZ and their multivariate correlations with symptoms using a multivariate regression approach. Overall, we found that these two sets of modes represent two abnormal dimensions of SCZ-specific features related to symptoms, allowing for independent measurement of brain scores and symptom scores on these two dimensions for each patient.

Mode 1 focuses more on negative and general scores, while mode 2 focuses more on positive scores. This seems to correspond to differences in the improvement of the three symptom domains after antipsychotic treatment: compared to positive symptoms, negative symptoms and general symptoms are less likely to improve [42]. It also suggests that the three types of symptoms may correspond to different neurodynamic bases. Our model only focuses on cortical regions, and positive symptoms are suggested to be related to interactions between the cortical and subcortical regions [43], which may explain why mode 2 has lower significance in PLS regression compared to mode 1. mode 1 exhibits a brain gradient from the limbic to low-level and then to high-level networks, where the top three loading networks correspond to the triple network hypothesis [44], indicating that the triple network hypothesis may represent a pathological dimension of SCZ.

Our study utilized transcriptomic GO enrichment analysis to provide a molecular perspective on the neurobiological basis of two PLS modes. We initially observed significant correlations between gene expression and brain loadings associated with both modes, supporting the specificity and heritability of SCZ from the standpoint of gene expression and micro-macro interactions [45]. This empirical genetic support could shed light on the etiology of SCZ. In the enrichment results, we identified several risk biological processes of SCZ previously reported in the literature, including “protein transport”, “synaptic signaling” and “neuron development” [46–49]. These biological processes exhibit mutual connections and influences. For instance, according to the “two-hit” hypothesis [49], early disruptions in neuron development caused by genetic or environmental factors lead to long-term vulnerability to a “second hit”, resulting in the onset of SCZ symptoms. The inter-cell signaling pathway in the brain may serve as one target for a “first hit” during early neuron development, potentially representing common underpinnings of SCZ. Meanwhile, “synaptic signaling” serves as one of the targets in the “two-hit” hypothesis, exerting a greater influence on mode 2, providing insights into its unique molecular mechanism. Overall, through a data-driven approach, we have established the association between microscale mechanisms and clinical symptoms via neurodynamic modeling, providing evidence that genetic influences on different biological processes may also contribute to the heterogeneity of SCZ.

### Limitations and future directions

Our study has several limitations. Firstly, although we included as many sites as possible and utilized scanners of different brands and models while ensuring consistency in scanning parameters, our approach based on a completely data-driven framework, remains a high demand for both quality and quantity of data in the dataset. Further validation in various larger datasets is needed to ensure its generalizability and applicability. Particularly, exploring the associations between these SCZ-specific features and clinical symptoms in longitudinal datasets holds potential for advancing their clinical applicability. Secondly, other neuroimaging modalities and multi-omics data may complement the refinement of neurodynamic models and the analysis of neurodynamic abnormalities in SCZ. For example, incorporating prior knowledge such as T1w/T2w map and RSFC gradient [8] into the state differential equations of the neurodynamic model to better fit the model; utilizing multimodal [50], cytoarchitectonic [51], or an individualized cortical parcellation [52] to better reflect the underlying structural organization; correcting the structural connectome for known biases to better characterize the dynamic interactions across brain regions. Thirdly, due to limitations in the size of the dataset, we did not examine whether the extracted SCZ-specific feature features are indeed SCZ specific, or whether some are comorbid factors of mental disorders. In the future, we can examine the expression and impact of these SCZ-specific features in other mental disorders through multi-disorder datasets. Lastly, the AHBA dataset was predominantly developed from Caucasian brains with a small and unevenly distributed sample size [25], which may not fully represent the physiology of the Chinese participants in our analysis. In the future, it will be essential to verify these findings by integrating multi-omics data from samples more closely matched in terms of gender, age, and ethnicity.

In summary, our study introduces a novel methodological framework that disentangle individual-level SCZ-specific spatiotemporal dynamic features in SCZ across the clinical, macro-, and microscale levels, advancing our understanding of the clinical and biological heterogeneity from the neurodynamic perspective. In the future, combining longitudinal samples, multimodal data, more effective and generalizable neurodynamic models, and multi-omics correlation analysis methods will allow for a deeper investigation into unraveling the heterogeneity of SCZ and providing targeted interventions and treatments.

## Supplementary information

Supplemental material

## Data Availability

Resting-state fMRI data preprocessing was implemented in an open MATLAB-based tool, BRANT (http://brant.brainnetome.org/en/latest). The code used for NeuroSynth meta-analysis is freely available at (https://www.github.com/gpreti/GSP_StructuralDecouplingIndex). Human gene expression data from the post-mortem tissues from six donors are available at (https://human.brain-map.org/static/download). The code for Spatial Autocorrelation (SA) spin test of cortical maps is freely available at (https://github.com/murraylab/brainsmash). The ToppGene (https://toppgene.cchmc.org) and abagen tools (https://github.com/rmarkello/abagen) which used to do the functional annotation of genes are all freely accessible. Our model and code are available at (https://github.com/wuyanwind/ndcvae).
