# Supplementary material for: Disorder-specific neurodynamic features in schizophrenia inferred by neurodynamic embedded contrastive variational autoencoder model
Source: Transl Psychiatry. 2024 Dec 18;14:496. doi: 10.1038/s41398-024-03200-7 (PMC11655856; doi:10.1038/s41398-024-03200-7)
Supplement: Supplementary file 1 — Supplemental material [file 41398_2024_3200_MOESM1_ESM.pdf]

Supplementary Material

Disorder-Specific Neurodynamic Features in Schizophrenia inferred by Neurodynamic Embedded Contrastive Variational Autoencoder Model

Table of Contents

Supplementary Methods 3
MRI acquisition. 3
MRI Preprocessing 3
Brain parcellations and canonical resting-state subnetworks 4
NeuroSynth term-based meta-analysis 4
Preprocessing pipeline of the gene expression matrix 5
Supplementary Table 6
Table S1. Demographic and clinical characteristics of schizophrenia participants, stratified according to site 6
Table S2. Correlations between SCZ-specific $\boldsymbol{\theta}_1^s$ and PANSS total, positive, negative and general scores after regressing out different control variables 7
Supplementary Figure 8
Figure S1. Evaluation of model optimization using a 5-fold cross-validation framework. 8
Figure S2. Detailed loss curves during the model optimization process. 10
Figure S3. Model optimization process and encoded parameters are robust to the use of brain parcellations. 11
Figure S4. Model parameters are robust to the use of group-level or individual-level structural connections 13
Figure S5. The effect of parameter space dimensionality on the model's performance and stability. 15
Figure S6. Evaluations of SCZ shared region-level parameters. 16
Figure S7. Evaluations of the hidden states of node systems *x*. 17
Figure S8. Feature dependencies within 44 time-series features. 19
Figure S9. NeuroSynth term-based meta-analysis. 20
Figure S10. The validation enrichment results for the top and bottom 1500 genes, selected based on the highest and lowest Pearson correlation coefficients between expression patterns and target brain maps. 21
Reference 22

Supplementary Methods

MRI acquisition.

The current study included resting-state fMRI, T1-weighted (T1w), and diffusion tensor imaging (DTI) data. T1-weighted images were acquired using a rapid gradient-echo sequence with the following parameters: matrix size = 256×256×192 (Siemens scanners) or 256×256×188 (GE scanners); voxel size = 1×1×1mm^3; inversion time (TI) = 1,100ms; slice thickness = 1mm. Resting-state fMRI data were acquired using an echo planar imaging (EPI) sequence with the following parameters: repetition time = 2,000ms; echo time = 30ms; flip angle (FA) = 90°; matrix size = 64×64; voxel size = 3.4375×3.4375×4.6mm^3; slice thickness = 4mm; gap between slices = 0.6mm; slices = 33; volumes = 240. The DTI data were acquired using a single-shot spin-echo EPI (SE-EPI) sequence from the participants of the PKU6 with the following parameters: matrix size = 128×128; voxel size = 2×2×3 mm^3 and without slice gap; FOV = 256×256 mm^2; slices = 50; 64 noncollinear diffusion gradient directions; 64 volumes with b-value = 1000 s/mm^2 and 1volume with b-value = 0 s/mm^2.

MRI Preprocessing.

Resting-state fMRI data obtained from our multi-site dataset underwent uniform preprocessing using BRANT version 3.35 [1]. The initial ten volumes were discarded to ensure magnetization stabilization. The preprocessing pipeline included the following steps: correction for slice timing, intra-participant realignment of EPI images, rigid-body transformation-based registration from T1-weighted (T1w) to the mean EPI image, spatial normalization of EPI images to the standard Montreal Neurological Institute (MNI) space, resampling of the normalized EPI images to a voxel size of 3×3×3 mm^3, removal of potential confounding effects through a multiple regression model, and temporal band-pass filtering within the frequency range of 0.01-0.08 Hz. Subsequently, the filtered signals were averaged within each brain region to obtain time series data for subsequent analyses.

The T1w data from our multi-site dataset were preprocessed using CAT12, an extension toolkit of SPM12, to calculate GMV. We further performed standardized preprocessing procedures, including denoising, intensity normalization, and linear and nonlinear registration using the MNI152 template. Subsequently, the segmented gray matter images were normalized and resampled to a voxel size of 1.5×1.5×1.5 mm^3. The normalized gray

matter images were subsequently modulated by multiplying the voxel values with the Jacobian determinant derived from spatial normalization and smoothed using a Gaussian kernel of $8 \times 8 \times 8$ mm^3 full-width at half maximum. Finally, the gray matter images were averaged within each brain region to obtain regional GMV for each participant.

The DTI data from participants at the PKU6 site (90 SCZ and 99 NC), underwent preprocessing to rectify distortions caused by head motion and eddy currents, and construct structural connectivity matrices for each participant. The group-level structural connections used in the neurodynamic model were obtained by averaging two groups of participants separately. The registration tools *flirt* and *fnirt* in FSL were employed to register from DTI space to T1 space and from T1 space to standard space. MRtrix toolbox [2] was employed to estimate the response functions by *dwi2response* tool, generate the tractogram with 1 million streamlines, and reconstruct white matter pathways and streamlines tractography. Due to limitations imposed by the neurodynamic model, we focused exclusively on cortical regions, and built the structural connectivity by mapping the reconstructed streamlines onto the Schaefer version-100 atlas.

Brain parcellations and canonical resting-state subnetworks.

The human brain regions were delineated into 100 cortical regions (Schaefer-100), originally derived from cortical intrinsic functional connectivity as developed by Schaefer et al. [3]. To characterize canonical resting-state subnetworks, we adopted the established canonical cortical parcellation, which consists of seven subnetworks initially introduced by Yeo et al. [4]. These subnetworks include visual (VIS), somatomotor (SOM), salience/ventral attention (SAL), dorsal attention (DAN), limbic (LIM), frontoparietal (FPN), and default mode (DMN). Each of the 100 cortical regions of interest (ROIs) from the functional parcellation is assigned to one of these resting-state canonical subnetworks according to the original developers. The Desikan-Killiany brain atlas, comprising 68 cortex regions (DK-68), was utilized for the robustness analysis.[5]

NeuroSynth term-based meta-analysis.

To decode the patterns of brain loading maps for significant PLS modes, we applied the Neurosynth database (https://neurosynth.org/), an online platform for large-scale meta-analysis of fMRI studies, to the two brain loading maps. We selected 24 topic terms that encompass a fairly comprehensive range of domains in behavior and cognition, as previously utilized in studies [6, 7]. We ranked and divided each brain loading map into 20

sections based on the magnitude of activation values. For each section, we created a new brain map by setting the values in the remaining regions to 0. We then weighted the z-scores of the topic terms with this sequence of values, reordered the topics, and visualized the results in a graph. Therefore, the topic terms activated in the first few columns are domains in behavior and cognition that are more closely related to the respective brain loading maps, and vice versa.

Preprocessing pipeline of the gene expression matrix

To construct gene expression matrix from the Allen Human Brain Atlas [8] transcriptome dataset, we employed the Abagen toolbox [9] to preprocess the gene expression data and map them to the 100 cortical parcellations in MNI space. Following Abagen's recommended protocol, our standardization preprocessing pipeline consisted of the following steps. First, we re-annotated the microarray probes using data provided by Arnatkeviciute et al., [9] and subsequently discarded probes that did not match a valid Entrez ID. Second, we removed probes with expression intensities lower than the background intensity in more than half of the samples across donors. Third, when multiple probes indexed the expression of the same gene, the probe with the most consistent regional variation pattern across the donors was selected. Next, the samples were assigned to the 100 cortical parcellation, if their MNI coordinates were within 2 mm of the given region. The samples that were not assigned to any region were discarded. To reduce the potential for misassignment, sample-to-region matching was constrained by hemispheric and gross structural divisions (i.e., cortical and subcortical regions). Gene expression values were normalized across samples within gross structural divisions using a robust sigmoid function and a unit interval rescale operation. Finally, by averaging the expression values of samples assigned to the same region separately for each donor and across donors, this workflow resulted in a regional gene expression matrix with 100 rows (representing brain regions) and 15,633 columns (representing the remaining genes).

Supplementary Table

Table S1. Demographic and clinical characteristics of schizophrenia participants, stratified according to site.

sites	PKU6	HLG	XIAN	XX_S	XX_G	WUHAN
**Number (SZ/NC)**	90/98	81/59	83/54	77/102	47/69	78/89
**Age (y) (SZ/NC)**	27.4 (6.7)/ 25.8 (5.3)	29.6 (8.7)/ 25.3 (5.4)	25.7 (6.4)/ 31.0 (6.9)	26.0 (5.3)/ 29.2 (7.2)	29.5 (7.5)/ 30.8 (7.2)	24.7 (4.7)/ 26.2 (6.3)
**Sex (M/F) (SZ/NC)**	56/34 53/45	32/49 31/28	46/37 31/23	40/37 53/49	28/19 35/34	28/50 46/43
**Duration (y) (SZ)**	4.7 (4.6)	5.9 (5.5)	1.8 (2.4)	3.2 (3.2)	3.9 (4.8)	4.0 (3.9)
**PANSS positive (SZ)**	23.7 (4.5)	26.2 (3.1)	23.0 (4.9)	24.5 (3.8)	22.8 (2.6)	23.8 (3.8)
**PANSS negative (SZ)**	18.2 (5.9)	16.5 (3.2)	22.7 (6.6)	23.9 (6.0)	19.5 (5.3)	21.2 (5.8)
**PANSS general (SZ)**	35.6 (5.4)	36.0 (4.0)	45.0 (8.5)	39.9 (6.2)	39.4 (5.4)	43.7 (7.5)
**PANSS total (SZ)**	77.5(9.9)	78.7(7.1)	90.8(15.3)	88.4(11.6)	81.8(8.3)	88.7(12.1)
**MR scanner (SZ/NC)**	Siemens Trio 3T	Siemens Trio 3T	Siemens Trio 3T	Siemens Verio 3T	GE Signa HDx 3T	GE Signa HDxt 3T
**Mean FD (mm) (SZ/NC)**	0.14 (0.07)/ 0.11 (0.04)	0.16 (0.11)/ 0.15 (0.08)	0.13 (0.07)/ 0.13 (0.07)	0.13 (0.08)/ 0.12 (0.06)	0.14 (0.07)/ 0.14 (0.08)	0.12 (0.10)/ 0.10 (0.05)

Table S2. Correlations between SCZ-specific $\boldsymbol{\theta_1^s}$ and PANSS total, positive, negative and general scores after regressing out different control variables.

Control variables	Statistics	PANSS scores			
		Total	Positive	Negative	General
NaN	*r*	**-0.187**	−0.034	**−0.167**	**−0.155**
	P	**5.8×10^{-5}**	0.47	**3.5×10^{-4}**	**8.8×10^{-4}**
Site	*r*	**-0.147**	−0.049	**−0.136**	**−0.105**
	P	**1.6×10^{-3}**	0.30	**3.5×10^{-3}**	**0.025**
Gender	*r*	**-0.189**	−0.008	**−0.167**	**−0.170**
	P	**3.2×10^{-5}**	0.91	**3.4×10^{-4}**	**2.5×10^{-4}**
Age	*r*	**-0.184**	−0.010	**−0.182**	**−0.150**
	P	**7.9×10^{-5}**	0.83	**9.5×10^{-5}**	**1.3×10^{-3}**
Education length	*r*	**-0.190**	−0.012	**−0.174**	**−0.167**
	P	**4.3×10^{-5}**	0.80	**1.9×10^{-4}**	**3.6×10^{-4}**
Age & gender	*r*	**-0.173**	−0.016	**−0.174**	**−0.136**
	P	**2.1×10^{-4}**	0.74	**1.9×10^{-4}**	**3.7×10^{-3}**
Site & age & gender & education length	*r*	**-0.146**	−0.054	**−0.144**	**−0.095**
	P	**1.7×10^{-3}**	0.25	**2.1×10^{-3}**	**0.043**

Supplementary Figure

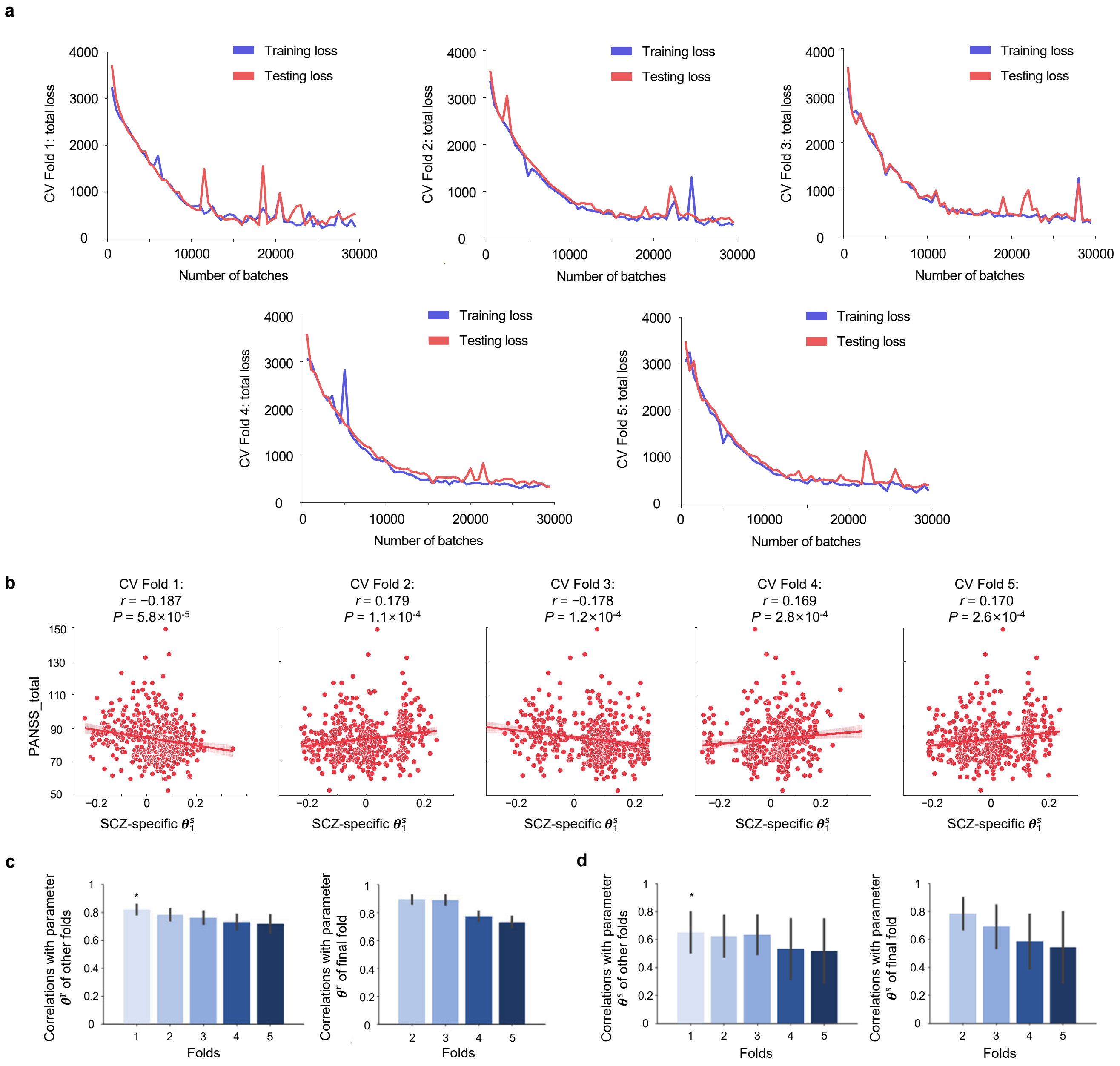

Figure S1. Evaluation of model optimization using a 5-fold cross-validation framework.

a, Training and testing loss curves for 5-fold cross-validation during the model optimization process. Each batch consists of 32 randomly selected individuals from both SCZ and NC groups. It is evident that after approximately 15,000 training batches, the total model loss stabilizes. The synchronized and consistent decrease in both the training and testing sets indicates the model's strong fitting performance on our dataset. **b,** Scatter plots depicting the associations between SCZ-specific $\boldsymbol{\theta}_1^{\mathrm{s}}$ of all five folds and PANSS total scores.

Significant correlations are observed between all five parameters and the PANSS total score, confirming the robustness of the model optimization. **c,** Bar plots illustrating the product-moment correlations of the encoded region-level parameters $\boldsymbol{\theta}^{\mathrm{r}}$ across the cross-validation folds. Fold 1, marked with an asterisk, was selected as the optimal training model due to its highest average correlation coefficient with other folds for the encoded parameters $\boldsymbol{\theta}^{\mathrm{r}}$ and $\boldsymbol{\theta}^{\mathrm{s}}$, indicating it as the most stable fold. Encoded parameters $\boldsymbol{\theta}^{\mathrm{r}}$ exhibited relative stability across the cross-validation folds, with product-moment correlations of $\boldsymbol{\theta}^{\mathrm{r}}$ across the cross-validation folds showing a mean ± SD of 0.76 ± 0.05. The product-moment correlations of $\boldsymbol{\theta}^{\mathrm{r}}$ with fold 1 across other cross-validation folds were mean ± SD of 0.82 ± 0.04. **d,** Bar plots representing the product-moment correlations of the encoded subject-level parameters $\boldsymbol{\theta}^{\mathrm{s}}$ across the cross-validation folds. The product-moment correlations of $\boldsymbol{\theta}^{\mathrm{s}}$ across the cross-validation folds were mean ± SD of 0.59 ± 0.18. The product-moment correlations of $\boldsymbol{\theta}^{\mathrm{s}}$ with fold 1 for other cross-validation folds showed a mean ± SD of 0.65 ± 0.15.

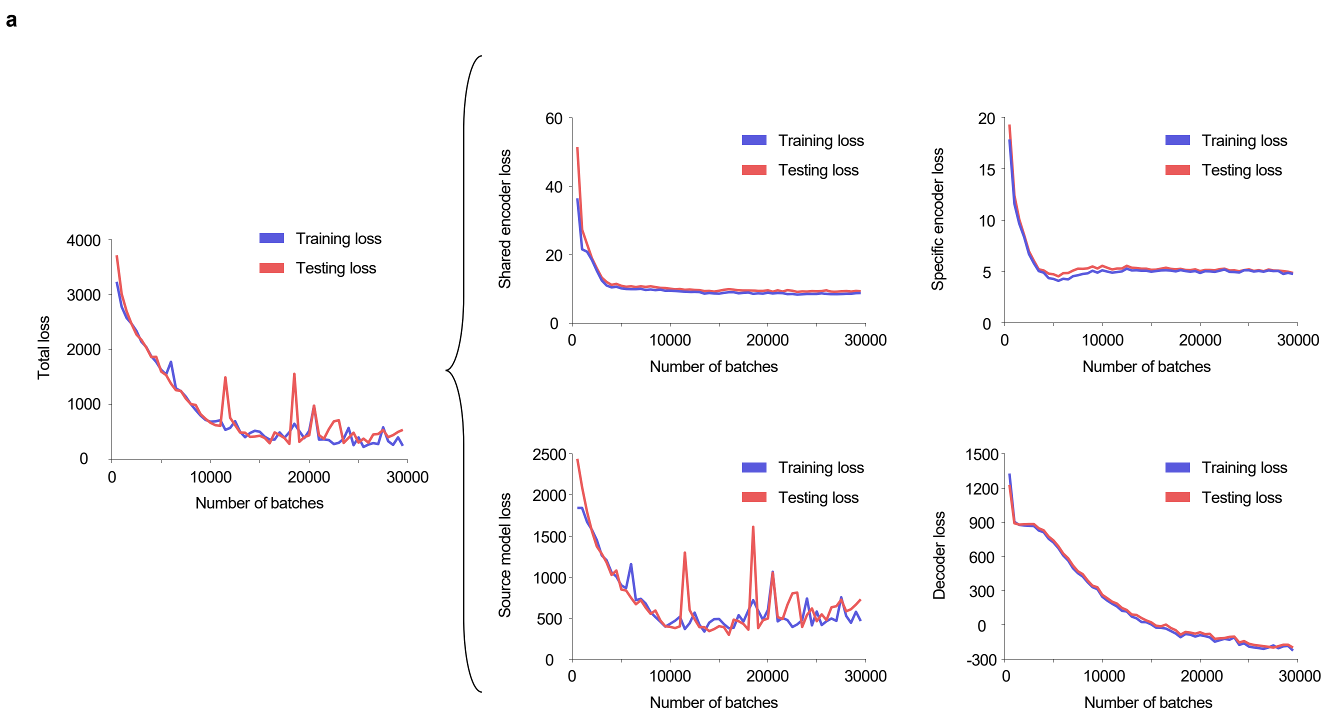

Figure S2. Detailed loss curves during the model optimization process.

This figure presents the detailed training and testing loss curves for the four components of the model: the shared encoder, the SCZ-specific encoder, the decoder, and the source model. Each batch comprises 32 SCZ and 32 NC samples randomly selected from their respective groups. The total loss of the encoders stabilizes after approximately 5,000 training batches, whereas the source model requires around 10,000 batches to reach stability. The decoder achieves stability after approximately 25,000 training batches. The synchronized and stable reduction in both training and testing loss across all four components underscores the model's robust fitting performance on our dataset.

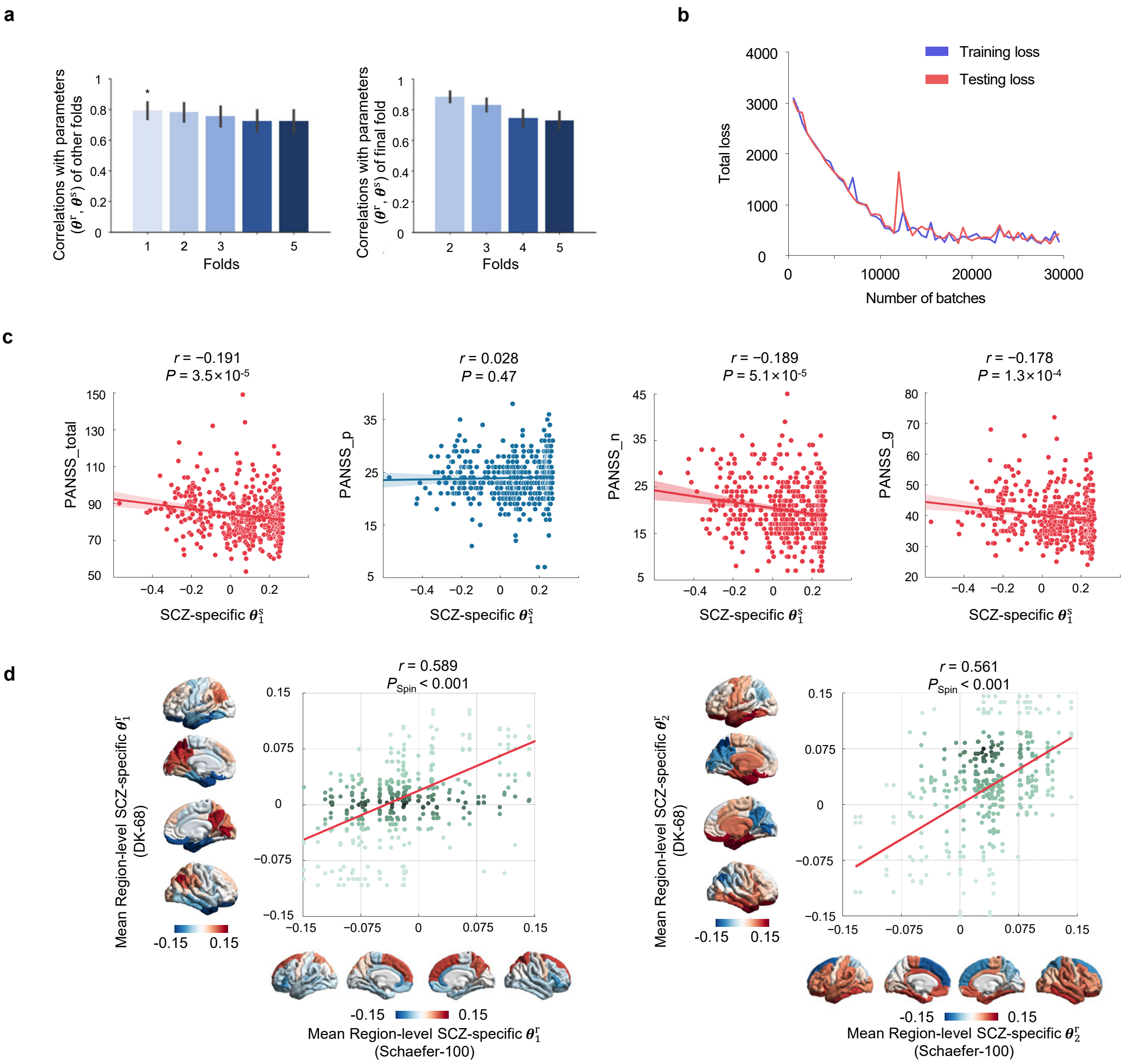

Figure S3. Model optimization process and encoded parameters are robust to the use of brain parcellations.

Control analysis was conducted using fMRI-based time series and dMRI-based structural connectivity (SC) data with the DK-68 parcellation, derived from the Desikan-Killiany anatomical atlas, which includes 68 cortical regions. The model's structure remained unchanged, except for adjusting the number of brain regions used for input and output. **a,** Bar plots illustrating the product-moment correlations of the encoded parameters $\boldsymbol{\theta}^{\mathrm{r}}$ and $\boldsymbol{\theta}^{\mathrm{s}}$ across the cross-validation folds. Fold 1, marked with an asterisk, was selected as the optimal training model due to its highest average correlation coefficient with other folds (with mean ± SD of 0.79 ± 0.06), indicating it as the most stable fold. Encoded parameters $\boldsymbol{\theta}^{\mathrm{r}}$ exhibited relative stability across the cross-

validation folds, with product-moment correlations across the cross-validation folds showing a mean ± SD of 0.75 ± 0.07. **b**, Training and testing loss curves for the optimal training model (fold 1) during the model optimization process. Each batch consists of 32 randomly selected individuals from both SCZ and NC groups. It is evident that after approximately 17,000 training batches, the total model loss stabilizes. The consistent decrease in both training and testing sets, similar to the results obtained using data with SCH-100 parcellation, underscores the resilience of the model optimization process across different parcellation schemes. **c**, Scatter plots showing the associations between SCZ-specific $\boldsymbol{\theta}_1^{\mathrm{s}}$ and PANSS total, positive, negative and general scores. **d**, Scatter plots illustrating the correlations between mean region-level SCZ-specific $\boldsymbol{\theta}^{\mathrm{r}}$ under two parcellation schemes. Region-level parameters were consistently mapped to the fsaverage5 surface space. The high correlations observed between the two parcellations confirm the robustness of encoded parameters to different parcellation schemes.

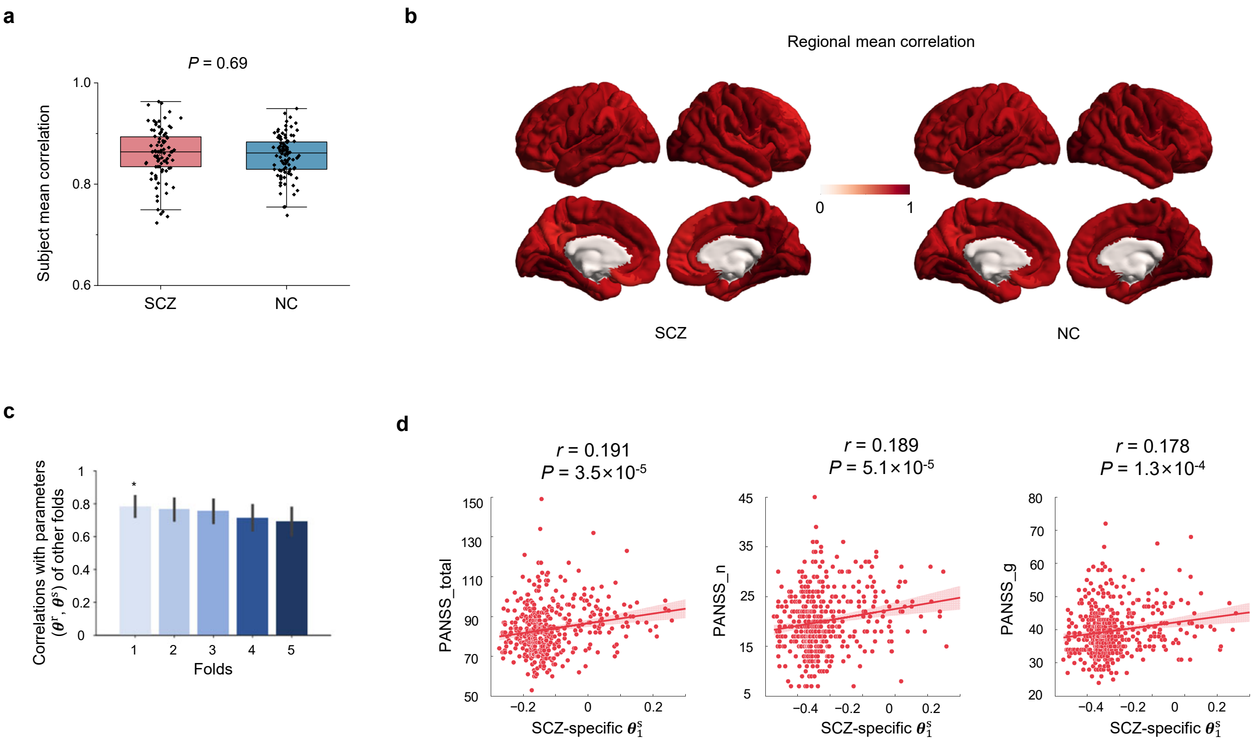

Figure S4. Model parameters are robust to the use of group-level or individual-level structural connections.

Control analysis was conducted using individual-level structural connections for samples at the PKU6 site, employing a 5-fold cross-validation framework. We first examined the correlation between the external inputs (u_{ext}) received by each brain region using individual-level and group-average structural connections. Subsequently, we retrained the model using sample data with individual-level structural connections and evaluated the resulting model parameters. **a,** Subject mean correlations between the external inputs (u_{ext}) received by each brain region using individual-level and group-average structural connections. The average correlation coefficient at the individual level is high (mean ± SD of 0.86 ± 0.05), with no significant difference between groups (*P*=0.69). **b,** Regional mean correlations between the external inputs (u_{ext}) received by each brain region using individual-level and group-average structural connections. The average correlation coefficient across all brain regions exceeds 0.8, indicating that the choice between individual or group-level SC had minimal impact on input data. **c,** Bar plots illustrating the product-moment correlations (mean ± SD of 0.74 ± 0.07) of the encoded parameters ($\boldsymbol{\theta}^{\text{r}}$ and $\boldsymbol{\theta}^{\text{s}}$) across the cross-validation folds. **d**, Scatter plots showing the significant correlations between SCZ-specific $\boldsymbol{\theta}_1^{\text{s}}$ and PANSS total, negative and general scores.

The results obtained using individual-level structural connections, which closely mirror those at the group level (i.e., stable model optimization and meaningful encoding parameters), indicate that brain dynamics are not significantly affected by minor variations in structural connections. This aligns with previous research, demonstrating that changes in structural connections do not alter dynamic metrics (functional connections) measured in patients, while changes in dynamic parameters (such as global coupling strength G) can shift the dynamics from the optimal healthy regime and account for the dysfunction observed in functional connections [10].

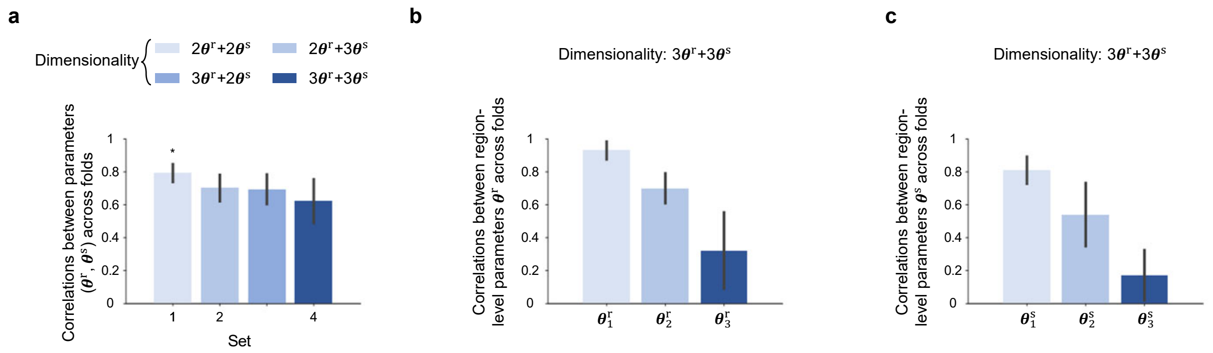

Figure S5. The effect of parameter space dimensionality on the model's performance and stability.

a, Bar plots illustrating the product-moment correlations of the encoded parameters ($\boldsymbol{\theta}^{\mathrm{r}}$ and $\boldsymbol{\theta}^{\mathrm{s}}$) across the cross-validation folds, using four different parameter space dimensionalities and a five-fold cross-validation framework. **b,** Bar plots illustrating the product-moment correlations between region-level parameters $\boldsymbol{\theta}^{\mathrm{r}}$ across folds using a parameter space dimensionality of $3\boldsymbol{\theta}^{\mathrm{r}}+3\boldsymbol{\theta}^{\mathrm{s}}$. **c,** Bar plots illustrating the product-moment correlations between region-level parameters $\boldsymbol{\theta}^{\mathrm{s}}$ across folds using a parameter space dimensionality of $3\boldsymbol{\theta}^{\mathrm{r}}+3\boldsymbol{\theta}^{\mathrm{s}}$. As the dimensionality of the parameter space increases from 2 to 3, a noticeable decline in model stability is observed. This is primarily due to a significant drop in the correlation coefficients between the third parameters ($\boldsymbol{\theta}_3^{\mathrm{r}}$, mean ± SD of 0.32 ± 0.21; $\boldsymbol{\theta}_3^{\mathrm{s}}$, mean ± SD of 0.17 ± 0.15) across folds when compared to those of the first two parameters ($\boldsymbol{\theta}_1^{\mathrm{r}}$, mean ± SD of 0.93 ± 0.05; $\boldsymbol{\theta}_1^{\mathrm{s}}$, mean ± SD of 0.81 ± 0.08; $\boldsymbol{\theta}_2^{\mathrm{r}}$, mean ± SD of 0.70 ± 0.09; $\boldsymbol{\theta}_2^{\mathrm{s}}$, mean ± SD of 0.54 ± 0.2). Although increasing the number of parameters may improve the model's fit to the training data, the instability and reduced generalization ability introduced by the third parameter suggest that we should focus on the results based on a parameter space dimensionality of 2 in our subsequent study.

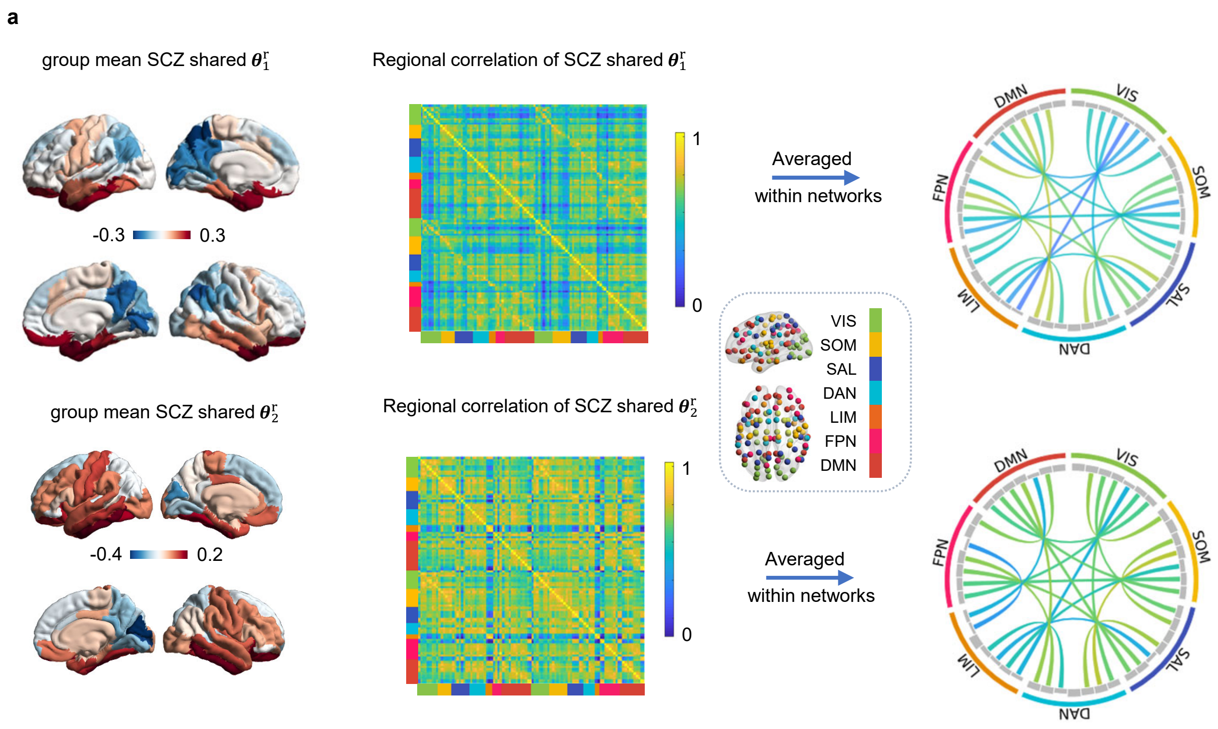

Figure S6. Evaluations of SCZ shared region-level parameters.

a, Mean SCZ shared region-level parameter $\boldsymbol{\theta}_1^{\mathrm{r}}$ and $\boldsymbol{\theta}_2^{\mathrm{r}}$ (100 regions) inferred by averaging the parameter matrix (456 subjects × 100 regions) on the subject dimension. **b,** Regional correlations of SCZ shared $\boldsymbol{\theta}_1^{\mathrm{r}}$ and $\boldsymbol{\theta}_2^{\mathrm{r}}$ (100 regions × 100 regions) inferred by taking the Pearson correlation coefficients of the parameter matrix (456 subjects × 100 regions) on the subject dimension.

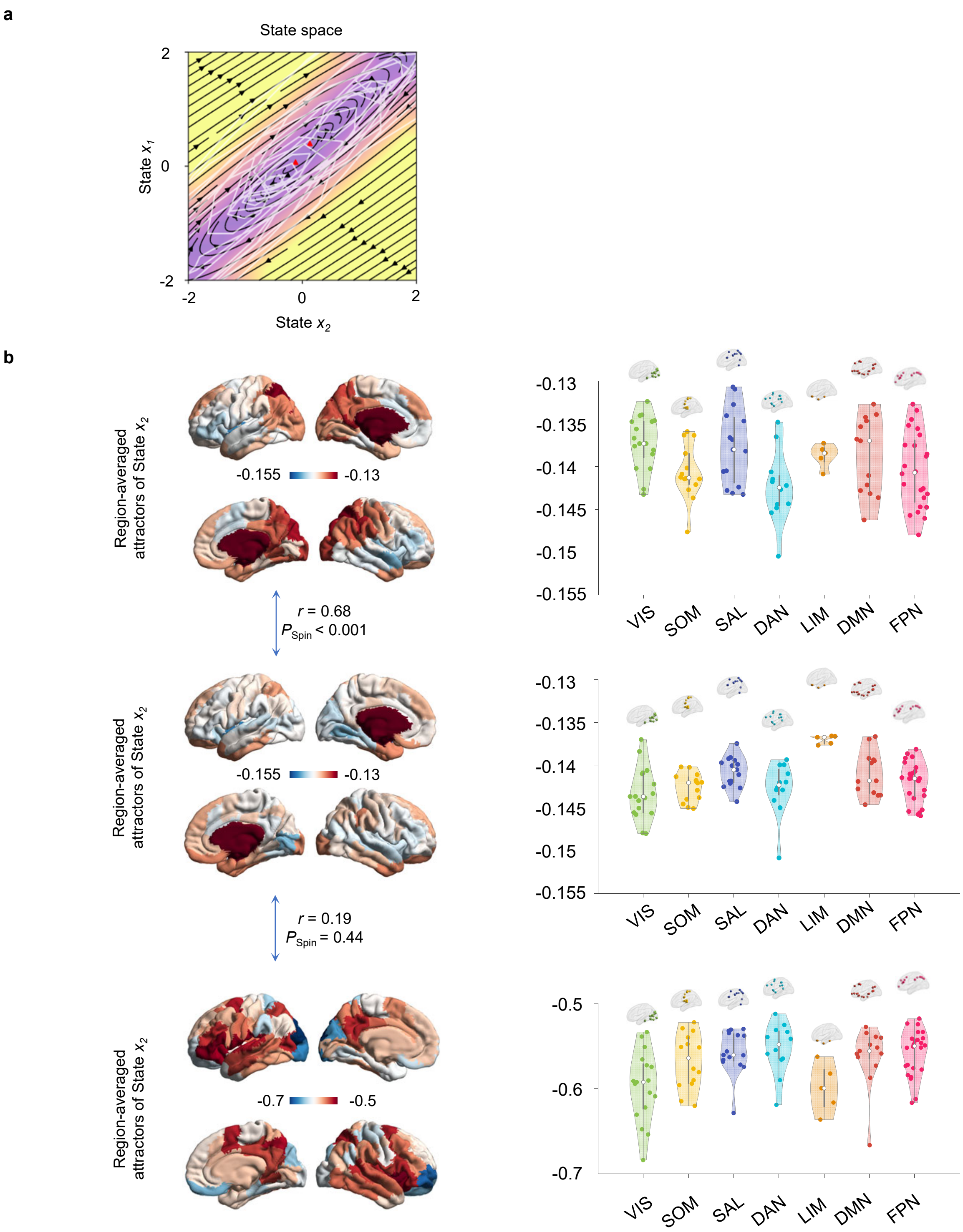

Figure S7. Evaluations of the hidden states of node systems *x*.

a, Among all samples, the only one brain region from one control individual with two fixed points, showing bistability. The vector field is assessed assuming zero network input and the inferred parameters. Background color indicates velocity magnitude; white lines indicate encoded time series of the node states; red triangles

indicate fixed points. The extracted states (white lines) exhibited oscillations around the two fixed points. **b,** Regional averaged attractors of state $\boldsymbol{x}_2$ (100 regions) inferred by averaging the attractor matrix of state $\boldsymbol{x}_2$ (471 NCs or 456 SCZs $\times$100 regions) on the subject dimension. From left to right are the shared state x_2 of the NC group, the shared state $\boldsymbol{x}_2$ of the SCZ group, and the specific state $\boldsymbol{x}_2$ of the SCZ group. The shared states of the NC group and the SCZ group exhibit similar brain gradient patterns, with the latter showing smaller inter-regional variances. However, the specific state $\boldsymbol{x}_1$ of the SCZ group present distinctive gradient patterns compared to both shared states.

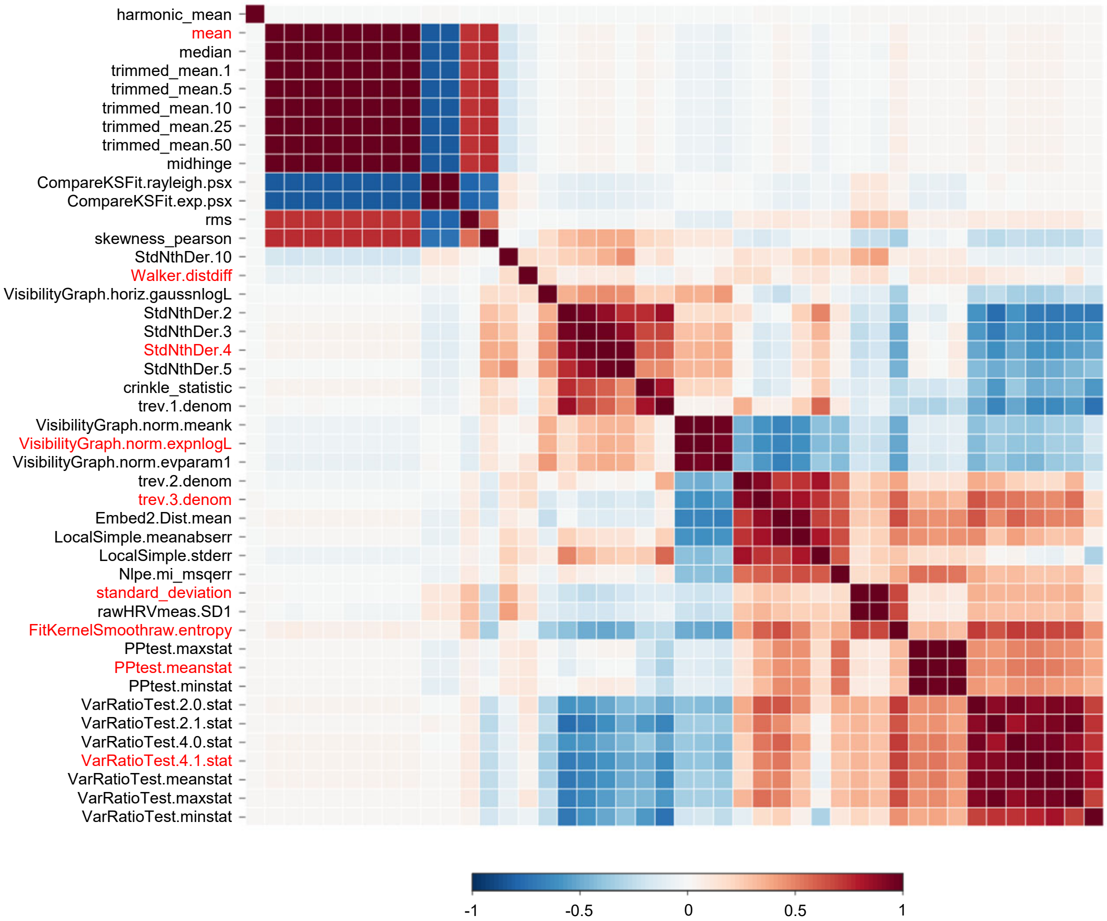

Figure S8. Feature dependencies within 44 time-series features.

These 44 time-series features encompass linear characteristics associated with data distribution, non-linear dynamics defined by entropy and autocorrelation, temporal non-stationarity assessed through the unit root method, and temporal stochasticity evaluated using the variance ratio test. We examined the Pearson correlation coefficients among these 44 features in SCZ-specific encoded states. In each cluster with high intercorrelation, we finally selected 9 representative features.

a

b

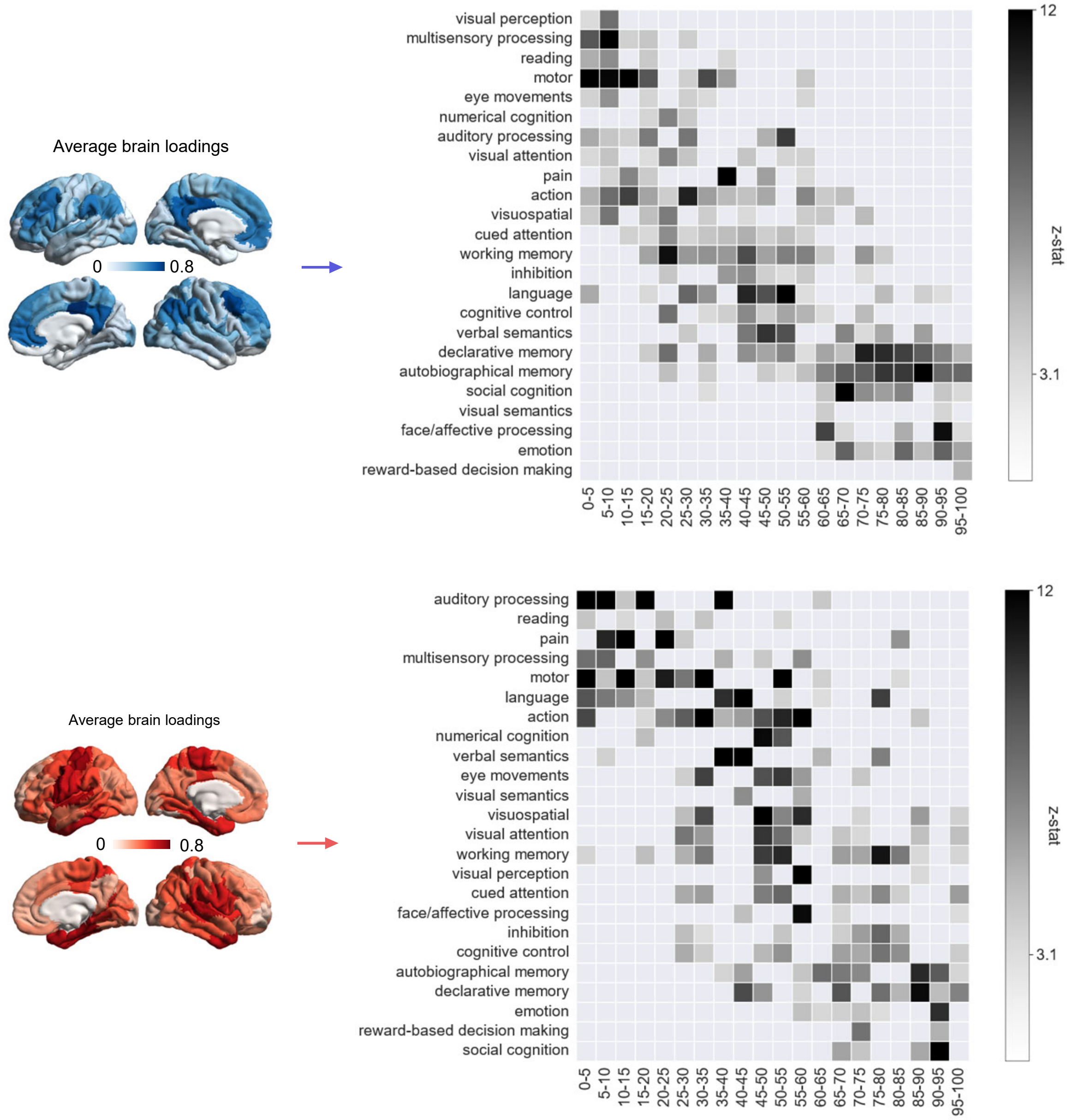

Figure S9. NeuroSynth term-based meta-analysis.

Each brain loading map was ranked and divided into 20 sections based on the magnitude of activation values. **a,** Terms of cognitive relevance for 20 sections of average brain loadings in mode 1. **b,** Terms of cognitive relevance for 20 sections of average brain loadings in mode 2. The topic terms activated in the first few columns are domains that are more closely related to the brain loading maps, and vice versa. Both modes showed robust associations with motor and multisensory processing, as well as action. Additionally, mode 2 demonstrated an additional influence on pain and auditory processing.

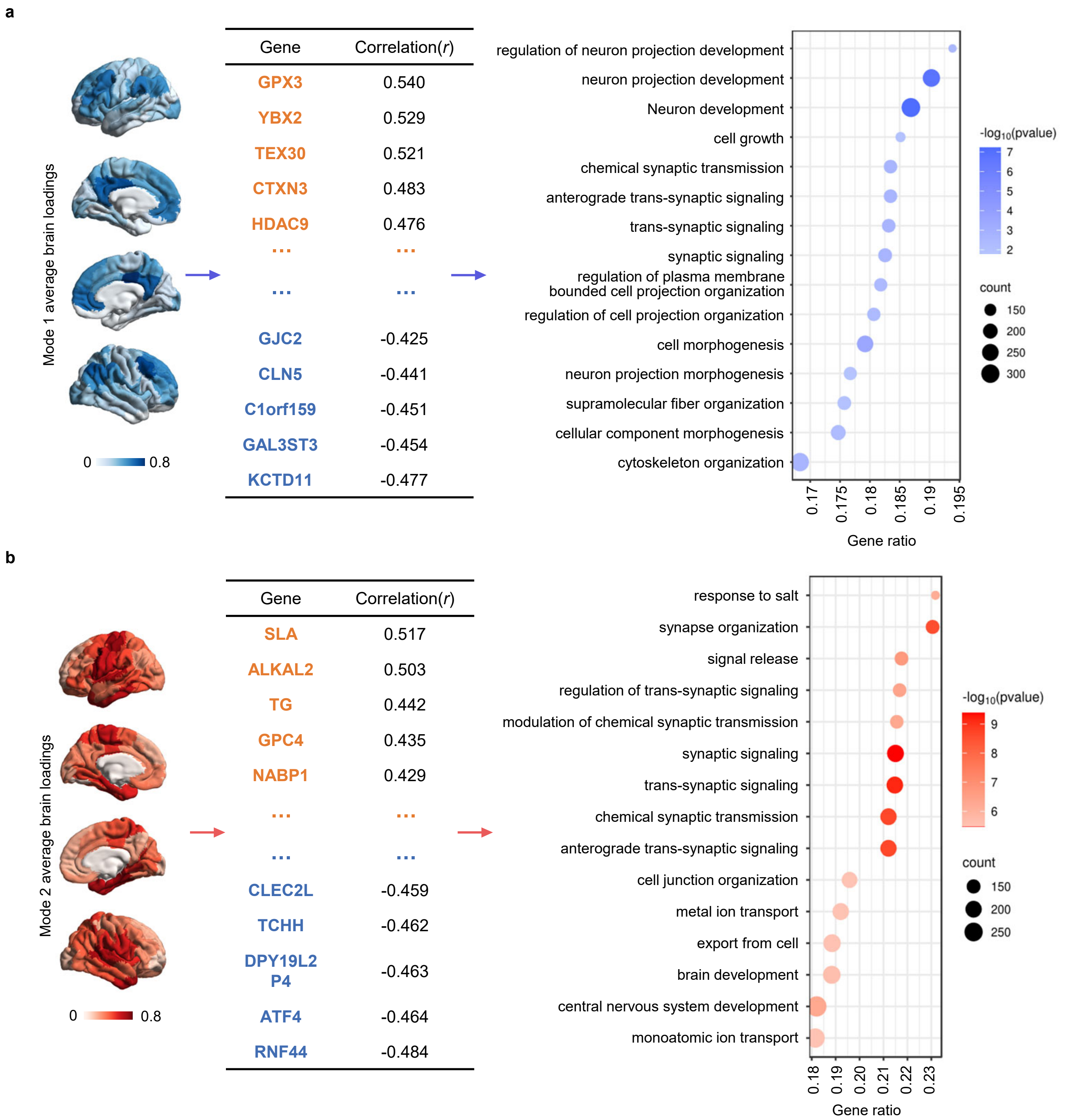

Figure S10. The validation enrichment results for the top and bottom 1500 genes, selected based on the highest and lowest Pearson correlation coefficients between expression patterns and target brain maps.

a, Enrichment results of average brain loadings in mode 1. **b,** Enrichment results of average brain loadings in mode 2.

Reference

1. Xu K, Liu Y, Zhan Y, Ren J, Jiang T. BRANT: a versatile and extendable resting-state fMRI toolkit. Frontiers in neuroinformatics. 2018;12:52.
2. Tournier J-D, Smith R, Raffelt D, Tabbara R, Dhollander T, Pietsch M, et al. MRtrix3: A fast, flexible and open software framework for medical image processing and visualisation. Neuroimage. 2019;202:116137.
3. Schaefer A, Kong R, Gordon EM, Laumann TO, Zuo X-N, Holmes AJ, et al. Local-global parcellation of the human cerebral cortex from intrinsic functional connectivity MRI. Cereb Cortex. 2018;28(9):3095-114.
4. Yeo BT, Krienen FM, Sepulcre J, Sabuncu MR, Lashkari D, Hollinshead M, et al. The organization of the human cerebral cortex estimated by intrinsic functional connectivity. J Neurophysiol. 2011.
5. Desikan RS, Ségonne F, Fischl B, Quinn BT, Dickerson BC, Blacker D, et al. An automated labeling system for subdividing the human cerebral cortex on MRI scans into gyral based regions of interest. Neuroimage. 2006;31(3):968-80.
6. Margulies DS, Ghosh SS, Goulas A, Falkiewicz M, Huntenburg JM, Langs G, et al. Situating the default-mode network along a principal gradient of macroscale cortical organization. Proc Natl Acad Sci. 2016;113(44):12574-9.
7. Preti MG, Van De Ville D. Decoupling of brain function from structure reveals regional behavioral specialization in humans. Nat Commun. 2019;10(1):4747.
8. Hawrylycz MJ, Lein ES, Guillozet-Bongaarts AL, Shen EH, Ng L, Miller JA, et al. An anatomically comprehensive atlas of the adult human brain transcriptome. Nature. 2012;489(7416):391-9.
9. Markello RD, Arnatkeviciute A, Poline J-B, Fulcher BD, Fornito A, Misic B. Standardizing workflows in imaging transcriptomics with the abagen toolbox. elife. 2021;10:e72129.
10. Cabral J, Fernandes HM, Van Hartevelt TJ, James AC, Kringelbach ML, Deco G. Structural connectivity in schizophrenia and its impact on the dynamics of spontaneous functional networks. Chaos: An Interdisciplinary Journal of Nonlinear Science. 2013;23(4).
